# Microenvironmental sensing by fibroblasts controls macrophage population size

**DOI:** 10.1073/pnas.2205360119

**Published:** 2022-08-05

**Authors:** Xu Zhou, Ruth A. Franklin, Miri Adler, Trevor S. Carter, Emily Condiff, Taylor S. Adams, Scott D. Pope, Naomi H. Philip, Matthew L. Meizlish, Naftali Kaminski, Ruslan Medzhitov

**Affiliations:** ^a^Department of Immunobiology, Yale University School of Medicine, New Haven, CT 06510;; ^b^Broad Institute of MIT and Harvard, Cambridge, MA 02142;; ^c^Department of Electrical Engineering and Computer Science, Massachusetts Institute of Technology, Cambridge, MA 02139;; ^d^Pulmonary Critical Care and Sleep Medicine, Yale University School of Medicine, New Haven, CT 06510;; ^e^HHMI, Yale University School of Medicine, New Haven, CT 06510

**Keywords:** macrophage, fibroblast, growth factor

## Abstract

Animal tissues comprise diverse cell types. However, the mechanisms controlling the number of each cell type within tissue compartments remain poorly understood. Here, we report that different cell types utilize distinct strategies to control population numbers. Proliferation of fibroblasts, stromal cells important for tissue integrity, is limited by space availability. In contrast, proliferation of macrophages, innate immune cells involved in defense, repair, and homeostasis, is constrained by growth factor availability. Examination of density-dependent gene expression in fibroblasts revealed that Hippo and TGF-β target genes are both regulated by cell density. We found YAP1, the transcriptional coactivator of the Hippo signaling pathway, directly regulates expression of *Csf1*, the lineage-specific growth factor for macrophages, through an enhancer of *Csf1* that is specifically active in fibroblasts. Activation of YAP1 in fibroblasts elevates *Csf1* expression and is sufficient to increase the number of macrophages at steady state. Our data also suggest that expression programs in fibroblasts that change with density may result from sensing of mechanical force through actin-dependent mechanisms. Altogether, we demonstrate that two different modes of population control are connected and coordinated to regulate cell numbers of distinct cell types. Sensing of the tissue environment may serve as a general strategy to control tissue composition.

## Significance

Collections of distinct cell types constitute animal tissues. To perform their unique functions, each cell type must exist in the correct number and proportion in a given tissue compartment. However, many of the mechanisms regulating and coordinating cell population sizes remain enigmatic. Our study characterizes two different modes of population size control, utilized by two ubiquitous cell types, macrophages and fibroblasts. Macrophage populations are more sensitive to the presence of growth factors in the environment and fibroblasts are more sensitive to space limitations. Intriguingly, space-sensing mechanisms in fibroblasts directly control the production of growth factor for macrophages and thus macrophage numbers. This link suggests a mechanism by which macrophage compartment size is controlled by stromal cells according to the microenvironment.

**Fig. 1.**
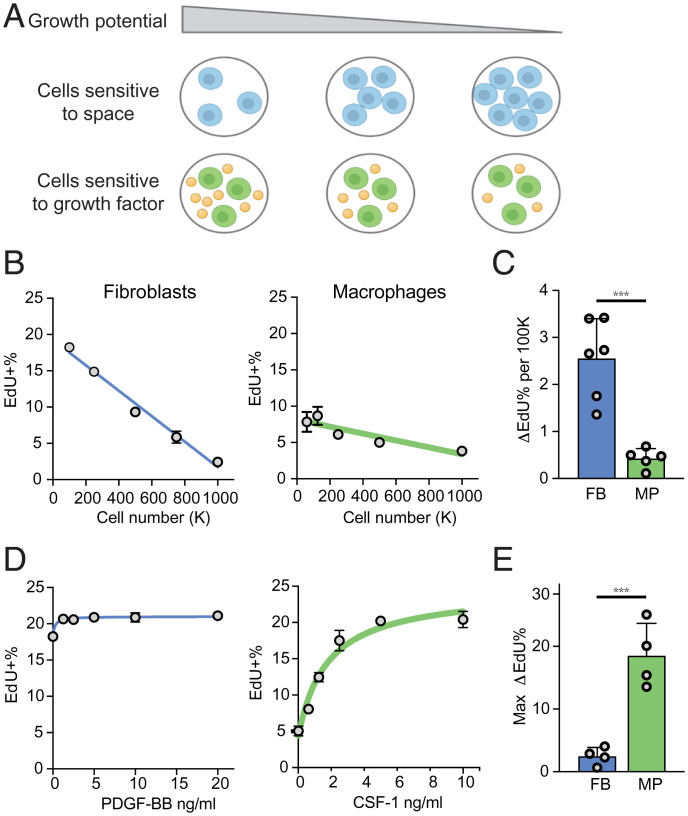
Fibroblasts and macrophages use two distinct mechanisms to control cell numbers. (*A*) Two proposed mechanisms of cell number control in a given tissue compartment. Orange spheres represent growth factors. (*B*) Proliferation of BMDMs and MEFs, measured as the percentage of EdU^+^ cells after 2 h of EdU labeling, following overnight culture at the indicated densities. (*C*) Cell density-dependent proliferation, estimated based on the linear fit of density-dependent growth as in *B* and quantified as the differential proliferation per 100,000 cells. Each dot represents an independent experiment. (*D*) Proliferation of BMDMs and MEFs stimulated with recombinant CSF-1 or PDGF-BB, respectively, measured as the percentage of EdU^+^ cells after 2 h of EdU labeling, cultured at the indicated growth factor concentration overnight. (*E*) Growth factor-dependent proliferation, quantified as the maximum change of EdU^+^ cells with the addition of growth factors **P < 0.001, t-test.

Animal tissues consist of multiple cell types present in appropriate numbers and ratios (1). Each cell type requires a specific growth factor for survival and proliferation, and therefore local availability of growth factors can control cell numbers within tissue compartments (2). However, growth factors alone are often insufficient to determine tissue compartment size (3). In fact, the maximum population size that can be supported in a particular environment (the carrying capacity of the environment) is determined by any limiting factor of that environment, such as space, nutrients, and oxygen levels (4). The interplay between growth factor availability and the carrying capacity of tissue environment in defining compartment size is not well understood. In particular, it is not well known how the numbers of different cell types within tissue compartments are maintained and coordinated (5). Factors that control compartment size, and ultimately tissue and organ size, generally fall into two categories (Fig. 1*A*).

First, compartment size can be controlled by the tissue microenvironment (6). One of the best understood examples is space availability, which is sensed through mechanical properties of the environment, including physical contact with the extracellular matrix (ECM) or neighboring cells (7–10). In this scenario, cells proliferate until available space is used up, at which point cell–ECM or cell–cell contacts suppress further cell division to reach a maximal cell number in a given environment. This space-dependent constraint can be sensed by signaling pathways, such as the Hippo signaling pathway (11–13) and mechanosensitive G protein-coupled receptors (14), and is reflected in an in vitro phenomenon of “contact inhibition” of growth (15). However, mechanosensing of space availability is not a usable strategy for some cell types, including hematopoietic cells, which are not constrained by space. Thus, a second strategy to control compartment size is through the availability of lineage-specific growth factors (2). Indeed, the numbers of naïve and memory T cells is maintained by interleukin (IL)-7 (16, 17) and the number of macrophages is limited by CSF1 (18–20). In these examples, the cell numbers depend on the local availability of appropriate growth factors, and therefore population size in a tissue compartment is limited by growth factor availability rather than space availability (21). According to this paradigm, if cell numbers are above or below the level that can be supported by available growth factors, cells will either die or proliferate, respectively, until they reach steady state (2).

Although for a given cell type either space or growth factor availability can be the dominant factor controlling cell numbers, any factor required for cell proliferation can impose a limit on population size. Thus, even growth of cell types normally restricted by space availability can become dependent on growth factor availability if the growth factor rather than space becomes a limiting factor. Indeed, some cell types can be limited by either space or growth factor availability, depending on the circumstances. For example, proliferation of hepatocytes is inhibited by Hippo-YAP signaling (22–24) while also regulated by hepatic growth factors (25). Intestinal epithelial cells are sensitive to mechanical forces sensed by PIEZO1 (14) and are also dependent on several growth factors, such as EGF and WNTs (26). Furthermore, different cell types within the same tissue compartment may employ different strategies to control each of their population numbers. It is unclear how cells using these two strategies coordinate their numbers within tissues. Since tissues are made up of multiple cell types, it is unknown whether sensing of environmental cues in one cell type would influence the population size of another cell type. Addressing these questions will be important for understanding of how different types of cells collectively constitute an organized and functional tissue.

Within tissues, growth factor for one cell type is typically produced by another cell type as a paracrine signal. Previously, we found that macrophages and fibroblasts, two cell types present in most mammalian tissues (20, 27), exchange growth factor signals PDGFB and CSF1 in vitro (18). This reciprocal communication through growth factors supports stable population ratios of these two cell types (18, 21, 28, 29). Similar paracrine production of CSF1 by fibroblasts for macrophages has been demonstrated in vivo in liver (30) and spleen (31). Here, we examined how different cell number control mechanisms apply to these two cell types, and found that at steady state, fibroblast proliferation is constrained by space availability, while macrophage proliferation is dependent on growth factor availability. Moreover, we found that sensing space availability in fibroblasts through YAP1 directly controls expression of the macrophage-specific growth factor CSF1. YAP1 activity in fibroblasts controls the absolute number of macrophages, as well as the ratios between these two cell types. We propose that the two strategies of cell number control, by space and growth factor availability, are inherently linked through the regulation of paracrine growth factor signals. This mechanism may provide a simple solution for automatically adjusting cell numbers and ratios within tissues.

## Results

### Fibroblasts and Macrophages Employ Different Modes of Compartment Size Control.

We previously observed that proliferation of fibroblasts, but not macrophages, is constrained by space availability: even in the presence of growth signals, space constraints set the carrying capacity of fibroblasts in vitro (18). We thus hypothesized that fibroblasts and macrophages may control cell numbers using different strategies. First, we formally examined whether fibroblast proliferation is more sensitive to space constraint, by measuring proliferation rates of bone marrow-derived macrophages (BMDMs, prototypical macrophages) and mouse embryonic fibroblasts (MEFs, prototypical fibroblasts) at different cell densities. The thymidine analog EdU is incorporated into newly synthesized DNA during S phase, allowing proliferating cells to be identified. The percentage of EdU^+^ cells in a short time window (2 h) was thus used as a proxy for the proliferation rate. Proliferation of fibroblasts was more density-dependent compared to proliferation of macrophages (Fig. 1 *B* and *C*). Second, we tested how growth rates of macrophages and fibroblasts depend on growth factor availability. We titrated the amounts of CSF1 for macrophages and PDGFB for fibroblasts and observed that addition of growth factors had a larger impact on proliferation of macrophages compared to fibroblasts (Fig. 1 *D* and *E*). Interestingly, even though proliferation of fibroblasts requires growth factors, in all conditions we have examined, space constraints regulate proliferation of fibroblasts almost five times more than the addition of growth factors; in contrast, growth factors influence proliferation of macrophages about five times more than changes in cell density. These data demonstrate two modes of compartment size control used by different cell types: responsiveness to environmental limitations, such as space availability, as observed for fibroblasts, and responsiveness to growth factor availability, as observed for macrophages.

These findings raise a question: If different cell types employ these distinct strategies to control their cell numbers, how is sensing of the environment and production of growth factors coordinated to achieve appropriate cellular composition of tissues? In a previous study, we found that macrophages and fibroblasts interact in a stable circuit via cell–cell contact and production of growth factors (18). While growth factors are required to maintain both populations, the stable number of fibroblasts is determined by space as the limiting factor. We thus hypothesized that these two distinct modes of population control do not operate independently, but rather are coupled and coordinated, to control cell numbers within tissues. Specifically, we hypothesized that sensing of space availability by fibroblasts is translated into growth factor production for macrophages, thereby adjusting cell numbers controlled by these two mechanisms.

### Fibroblasts Display Density-Dependent Gene-Expression Programs.

**Fig. 2.**
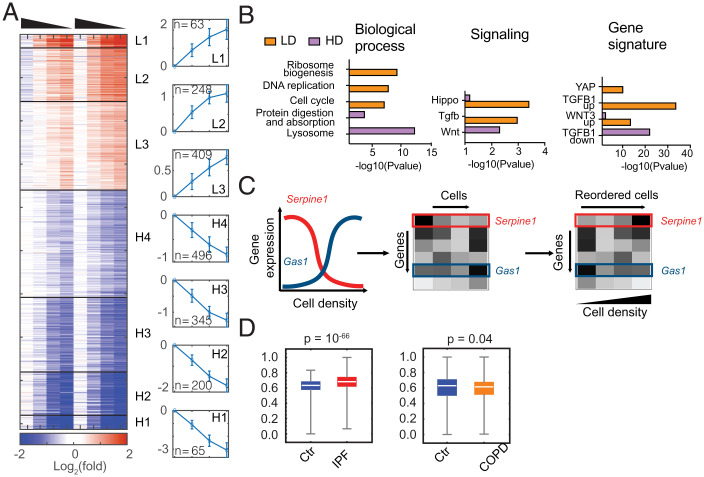
Fibroblasts exhibit density-dependent gene expression. (*A*) Heatmap showing relative expression of density-dependent genes in MEFs from two sets of biological replicates. Gene expression (TPM) is normalized to the average of two replicates at 10K density and shown after log_2_ transformation. Genes are clustered into seven groups using unsupervised K-mean clustering, organized from the most induced at low density to the most induced at high cell density. The average log_2_ fold-change of each cluster and the number of genes is shown on the right. Density of cells is indicated by the triangle above the heatmap, high to low. (*B*) Functional enrichment analyses of biological processes, Kyoto Encyclopedia of Genes and Genomes (KEGG) signaling pathways, and molecular signatures for genes induced in low density (LD) or high density (HD). (*C*) Using landmark genes that are density-dependent, cells in single-cell gene-expression data can be reordered along a pseudo density axis to find new density-dependent gene programs. (*D*) Violin plots showing the distribution of calculated cell density scores for single-cell RNA-seq of human fibroblasts extracted from IPF+control and COPD+control groups.

To test this hypothesis, we first evaluated cell density-dependent gene expression in fibroblasts. RNA sequencing (RNA-seq) was performed on fibroblasts at four cell densities, using two batches of separately isolated primary fibroblasts. The highest cell density is close to the theoretical carrying capacity estimated previously (highly confluent) (18), and the lowest cell density is close to the threshold at which fibroblasts cannot survive in monoculture (sparse). At each cell density, gene expression of biological replicates is highly correlated (*SI Appendix*, Fig. S1*A*). To select bona fide density-dependent genes, we determined differentially expressed genes, taking into account biological variation, and developed an automated algorithm to identify the genes with consistent expression changes across two datasets (*Materials and Methods* and *SI Appendix*, Fig. S1 *B* and *C*). In total, we found 1,826 genes to be highly expressed (transcripts per million [TPM] > 2) and significantly regulated by cell density in fibroblasts. Using unsupervised K-mean clustering, these genes were grouped into seven groups with distinct patterns of gene expression (Fig. 2*A*). Three clusters were up-regulated at low cell density (L1 to L3), and four clusters were up-regulated at high cell density (H1 to H4). L1 and H1 contained genes that are the most differentially regulated.

To understand the functions of the density-dependent expression programs, we performed enrichment analysis for these clusters independently and in combination. Genes induced at low cell density are enriched for ribosome biogenesis, cell cycle, DNA replication, and purine and pyrimidine metabolism, consistent with higher anabolic activity of proliferating cells at low cell density. In contrast, genes up-regulated at high cell density are enriched for genes associated with lysosomal function, ECM, and protein digestion and absorption (Fig. 2*B* and *SI Appendix*, Fig. S1*D*). Signaling pathways, including MAP kinase signaling, Hippo-YAP signaling, TGF-β signaling, Ras, vascular endothelial growth factor (VEGF), tumor necrosis factor (TNF), and IL-17 signaling pathways are significantly enriched at low cell density, and the Wnt signaling pathway is enriched at high cell density (Fig. 2*B* and *SI Appendix*, Fig. S1*E*). To further test if these signaling pathways may regulate the cell density-dependent expression programs, we examined the enrichment of gene-expression signatures curated in the Molecular Signature database among density-dependent clusters (32, 33). Hippo-YAP, TGF-β, and Wnt activation demonstrate the strongest enrichment for genes induced at low cell density, and genes repressed by TGF-β show the strongest enrichment at high cell density (Fig. 2*B* and *SI Appendix*, Fig. S1*F*). Overall, functional enrichment analysis revealed that Hippo-YAP and TGF-β signaling pathways are most highly correlated with density-dependent gene expression.

Space limitation in vitro in tissue culture dishes is distinct from space limitation within tissues due to the complexity of tissue microenvironment (34). We sought to test if the density-dependent expression programs we identified in vitro suggest similar mechanisms of cell density-sensing in physiological or pathological conditions. Fibrosis is characterized by excessive fibroblast proliferation (35). Recently, single-cell data of lung fibrosis samples from humans have become available (36). Inspired by previous work that reconstructed the spatial environment of single cells based on gradient expression (37, 38), we developed a computational algorithm to estimate “cell density” for individual cells based on their relative expression of density-dependent genes (Fig. 2*C*). This allowed us to compare the predicted cell density of fibroblasts between healthy individuals and patients with idiopathic pulmonary fibrosis (IPF) or chronic obstructive pulmonary disease (COPD) (39). Intriguingly, fibroblasts isolated from IPF patients display an increased tendency for high cell density (Fig. 2*D* and *SI Appendix*, Fig. S2*A*), consistent with the known invasive expansion of myofibroblasts (40). On the other hand, fibroblasts from COPD patients seem to be largely similar to healthy individuals (Fig. 2*D* and *SI Appendix*, Fig. S2*B*). These results suggest that the density-dependent expression programs identified in vitro may represent changes in fibroblasts that occur during fibrotic disease.

### Space Availability Modulates the Hippo-YAP and TGF-β Signaling Pathways.

The Hippo signaling pathway can be activated by diverse cellular and environmental signals and converges on a pair of homologous transcription factors YAP1 and TAZ (41). When appropriate environmental signals are present, such as cell–cell contact, YAP1 is excluded from the nucleus and either sequestered or degraded. When Hippo signaling is off, YAP1 translocates into the nucleus, where it forms a complex with the TEAD family DNA-binding transcription factors to regulate target gene expression. TGF-β signaling is activated by TGF-β family members, often bound to ECM in a latent form, that become activated through protease, integrin, or other processing events (42). Activation of TGF-β receptors leads to phosphorylation of receptor-regulated SMADs (R-SMADs), particularly SMAD2 and SMAD3. Phosphorylated R-SMADs translocate into the nucleus, forming a complex with SMAD4 to regulate target gene expression (43).

**Fig. 3.**
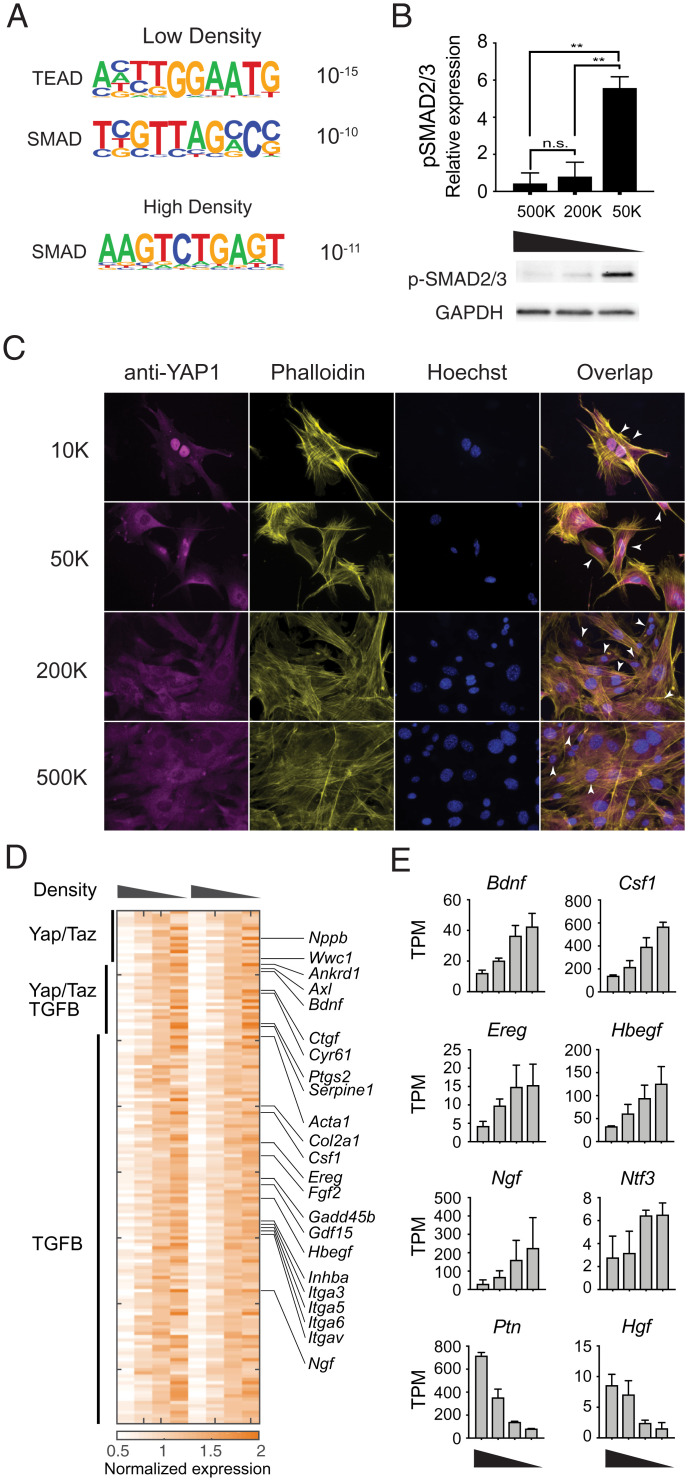
YAP1 and SMADs are activated in a density-dependent manner in fibroblasts. (*A*) Significantly enriched transcription factor binding motifs in genes induced at low or high density. (*B*) Phosphorylation of SMAD2/3 at different cell densities. (*Upper*) Quantification of phospho-Smad2/3 Western blot staining, normalized to GAPDH staining of the same sample, from three independent experiments. (*Lower*) Representative image of phospho-Smad2/3 and GAPDH Western blots. (*C*) Representative immunofluorescent images showing YAP1 localization in MEFs cultured at the specified cell densities overnight. Arrowheads denote examples of nuclear localization of YAP1. (*D*) Heatmap showing annotated targets of Hippo-YAP signaling and TGF-β signaling pathways at different cell densities. (*E*) Expression of representative growth factors significantly regulated by density (high to low density) **P < 0.01, t-test.

We found that both TEAD and SMAD binding sequence motifs are significantly enriched in the promoters of genes up-regulated at low cell density (Fig. 3*A*), while only SMAD binding motifs are enriched for genes induced at high cell density (Fig. 3*A*). SMAD proteins can act as both transcriptional activators and repressors (44). Distinct SMAD motifs enriched among the genes induced at either high or low densities suggest that cooperation with other transcriptional coactivators or corepressors may determine density-dependent activation or repression in addition to TGF-β signaling.

Next, we wanted to confirm whether the activities of the Hippo-YAP and TGF-β signaling pathways are regulated by cell density. Significantly increased phosphorylation of SMAD2/3 was observed at low cell density, suggesting that activation of SMAD proteins contribute to the observed density-dependent transcription programs (Fig. 3*B* and *SI Appendix*, Fig. S3*A*). To examine the role of Hippo-YAP signaling in density-dependent gene expression programs, nuclear localization of YAP1 was used to infer activity of the Hippo-YAP pathway. As expected, YAP1 is localized to the nucleus at low cell density. As cell density increases, YAP1 is gradually excluded from the nucleus, as indicated by the decrease in nuclear staining (Fig. 3*C*). Collectively, these data demonstrate that the activity of both TGF-β signaling and Hippo-YAP signaling are regulated in fibroblasts in a cell density-dependent manner, in response to space limitation. Indeed, several genes that are known to be directly controlled by YAP1/TAZ and SMAD proteins display density-dependent gene expression in fibroblasts. These genes include YAP1/TAZ targets—such as *Nppb*, *Akred1*, *Bdnf*, *Ctgf*, and *Cyr61*—as well as TGF-β target genes, such as *Serpine1*, *Acta1*, *Col2a1*, *Hbegf*, and *Ngf* (Fig. 3*D*).

### YAP1 and TGF-β Signaling Control Expression of Different Growth Factors in Response to Space Limitation.

Within the group of genes regulated by space limitation, we observed several growth factors specific for different cell lineages (*SI Appendix*, Fig. S3*B*). For example, neurotrophic growth factors (*Bdnf*, *Ngf*, *Nif3*, *Ptn*), epidermal growth factors (*Ereg*, *Hbegf*), hepatocyte growth factor *Hgf*, and myeloid growth factor *Csf1*, all show density-dependent expression patterns (Fig. 3*E*). In addition, we found that chemokine genes—including *Ccl8*, *Cxcl14* and *Cxcl15*, and the cytokine gene *Il33*—are differentially regulated by cell density (*SI Appendix*, Fig. S3*C*). In particular, the expression of *Csf1* was inversely related to fibroblast density, suggesting that density sensing by fibroblasts is coupled with *Csf1* production for macrophages (18).

**Fig. 4.**
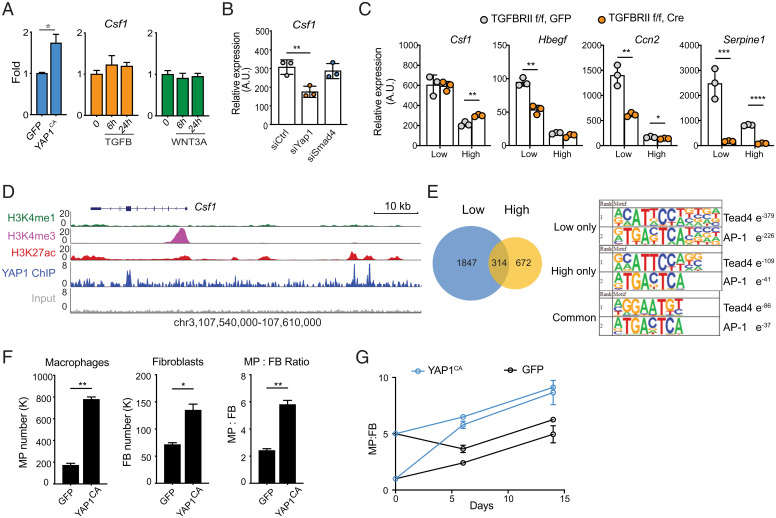
YAP1-dependent regulation of *Csf1* in fibroblasts controls macrophage numbers. (*A*) *Csf1* expression in MEFs carrying Yap^CA^ (*Left*), or treated with recombinant TGF-β (*Center*) or WNT3A (*Right*). MEFs were isolated from YapKI^fl/fl^ mice and then transduced with lentivirus carrying GFP or Cre-GFP (constitutively-active, YAP^CA^). (*B*) *Csf1* expression in MEFs 3 d after transfection with siRNAs targeting *Yap1*, *Smad4*, or scrambled control siRNA (siCTRL). (*C*) Expression of selected genes in wild-type and *Tgfbr2* knockout (KO) MEFs cultured at low or high density. Wild-type and *Tgfbr2* KO MEFs are generated by viral transduction of *Tgfbr2*^fl/fl^ MEFs with GFP or Cre-GFP vectors, respectively. (*D*) Genomic tracks displaying ChIP-seq occupancy of histone modifications (H3K27ac, H3K4me1, and H3K4me3) and endogenous YAP1 binding at the *Csf1* gene locus in MEFs. (*E*) Venn diagram showing the number of YAP1 binding peaks at low and high cell densities determined by ChIP-seq. The top ranked enrichment of transcription factor motifs is shown for peaks at high, low, or both cell densities. (*F* and *G*) Wild-type (GFP) and Yap^CA^ MEFs were plated together with WT BMDMs. Their numbers and ratios were determined by flow cytometry 6 d after coculture (*F*). BMDMs and MEFs were plated at different starting ratios and quantified at days 6 and 14 (*G*) *P < 0.05, **P < 0.01, ***P < 0.001, ****P < 0.0001.

To examine which pathway controls the expression of *Csf1* in fibroblasts, we tested whether activation of YAP1, TGF-β, or Wnt signaling is sufficient to regulate *Csf1* expression. Recombinant TGF-β or WNT3A do not induce the expression of *Csf1* at the mRNA level (Fig. 4*A*). However, *Hbegf* and *Ctgf* are induced by TGF-β, and to a lesser degree by WNT3A (*SI Appendix*, Fig. S3*D*). Next, we tested if activation of YAP1 can control *Csf1* expression. To activate YAP1 in primary fibroblasts, we used a previously established genetic model with constitutively active YAP1 (24). A mutation from Serine to Alanine prevents phosphorylation at residue 112 of YAP1 and results in constitutive nuclear localization. Overexpression of a similar mutant in the murine liver causes an increase in liver size, consistent with the known role of YAP1 in organ size control (45). In our model, *Yap1^S112A^-IRES-GFP* is expressed at the *Rosa26* locus, downstream of a floxed transcriptional STOP cassette. After introducing murine stem cell virus (MSCV) containing Cre recombinase, primary fibroblasts carrying the *Yap1^S112A^* allele express constitutively active YAP1 (referred to as YAP1^CA^). We found that the expression of *Csf1* is significantly elevated in these cells, in comparison to primary fibroblasts transduced with MSCV carrying GFP alone (Fig. 4*A*).

These data suggest that *Csf1* is induced by the activation of YAP1, but not TGF-β or Wnt signaling. However, overexpression of a constitutively active transcription factor may not faithfully reflect the role of endogenous YAP1 in response to space limitation. Therefore, we used small-interfering RNA (siRNA) knockdown to validate the functions of endogenous YAP1. Indeed, knocking down *Yap1* reduced *Csf1* expression by approximately twofold, similar to the difference between low and high cell densities, while knockdown of *Smad4*, the central transcription factor in TGF-β signaling, had minimal effects (Fig. 4*B*). Conversely, siRNA knockdown of *NF2*, an upstream suppressor of YAP1/TAZ, resulted in increased *Csf1* expression (*SI Appendix*, Fig. S3*E*). These data demonstrate that activation of endogenous YAP1 is necessary and sufficient to control *Csf1*. Both YAP1 and TAZ are transcriptional activators downstream of Hippo-YAP signaling. They interact with TEAD transcription factors, bind to similar DNA sequence motifs, and are both expressed in fibroblasts (*SI Appendix*, Fig. S3*F*). Furthermore, siRNA against *Wwrt1* (the gene encoding TAZ protein) had a partial effect on *Csf1* expression but targeting *Wwrt1* and *Yap1* simultaneously had no additive effect compared to targeting *Yap1* alone (*SI Appendix*, Fig. S3*E*). This suggests that YAP1 is the primary transcriptional activator controlling *Csf1* expression. However, it is worth noting that YAP1 and TAZ have differential effects on controlling the expression of other genes. These results are not due to differences in siRNA targeting efficiency, as 90% knockdown was achieved for all targets at the level of mRNA expression (*SI Appendix*, Fig. S3*G*). Finally, using *YAP1^CA^* fibroblasts, we compared gene expression of a subset of density-dependent growth factors to WT fibroblasts and identified additional growth factors regulated by YAP1 activity (*SI Appendix*, Fig. S3*H*). Expression of these growth factors also depends on endogenous levels of YAP1, as siRNA knockdown of *Yap1* reduces their expression (*SI Appendix*, Fig. S3*I*).

To further evaluate the TGF-β pathway in density-dependent growth factor expression, we used genetic and pharmacological targeting strategies. To genetically target TGF-β signaling, we isolated fibroblasts from *Tgfbr2*^fl/fl^ mice (46), transduced them with either Cre-GFP or GFP viral vectors, and sorted them for GFP-positivity to indicate successful transduction. In these cells deficient for TGFBR2, the main signaling receptor for TGF-β family ligands, expression of *Csf1* was unaffected (Fig. 4*C*). In contrast, *Hbegf*, *Ctgf*, and *Serpine1*, genes regulated by cell density and TGF-β signaling, were significantly reduced or entirely abolished (Fig. 4*C*). Pharmacological inhibition of the TGF-β receptor achieved similar results (*SI Appendix*, Fig. S3*J*). Using both genetic and pharmacological approaches, we demonstrated that signaling through TGFBR2 is responsible for density-dependent regulation of a subset of growth factors, including *Hbegf* and *Ctgf*. In contrast, the expression of *Csf1* was found to be controlled by YAP1, independent of TGF-β signaling.

### YAP1 Regulates the Expression of *Csf1* via a Conserved Distal Enhancer.

We next asked how the Hippo pathway regulates *Csf1* expression. *Csf1* is not known to be a YAP1 target gene and its promoter lacks a binding sequence for TEAD transcription factors. We speculated that YAP1 may regulate the expression of *Csf1* through distal regulatory elements. To test this hypothesis, we performed chromatin immunoprecipitation-sequencing (ChIP-seq) of endogenous YAP1 proteins in fibroblasts and discovered two distinct binding sites at 33 and 36 kb upstream of the *Csf1* transcription start site (Fig. 4*D*). Analysis of histone modifications from the mouse ENCODE project (47, 48) indicated that both YAP1 binding peaks are within regions that are enriched for H3K27ac but lack H3K4me1 and H3K4me3 marks, suggesting that YAP1 physically occupies two active distal enhancers of *Csf1* (Fig. 4*D*). On the other hand, we observed SMAD3 binding coincident with H3K27Ac at the *Hbegf* locus in fibroblasts (49), supporting both published data and our observations that *Hbegf* is induced downstream of TGF-β (*SI Appendix*, Fig. S4*A*). At the global level, we observed approximately three times more YAP1 binding events at low density compared to YAP1 binding events at high density (Fig. 4*E*). Both TEAD4 and AP-1 motifs were identified as highly enriched in YAP1 peaks, yet this enrichment was more robust at low density (Fig. 4*E*). This is consistent with previous work showing that YAP/TAZ, TEADs, and AP-1 family members cooperate to regulate gene expression (50).

Near the center of the +30-kb YAP1 peak at the *Csf1* gene, we identified two sequences matching the consensus binding motifs of TEADs. Genomic sequence alignment of this region demonstrated high conservation of these sites between a variety of mammalian species, including opossum, dog, rat, rhesus macaque, and human (*SI Appendix*, Fig. S4*B*). These data suggest that the mechanism by which Hippo-YAP signaling regulates *Csf1* expression may be conserved in mammals. Based on H3K27ac data from the human ENCODE project (47, 48), we identified a similar enhancer ∼30 kb upstream of the human *Csf1* gene that contains the conserved TEAD binding sites. Interestingly, the activity of this 30-kb enhancer is cell type-specific, in that the H3K27Ac mark is observed in fibroblasts, but not in endothelial or myeloid cells (*SI Appendix*, Fig. S4*C*). Conversely, an enhancer ∼12 kb upstream of the transcription start site, which has the AP-1 but lacks the TEAD binding motifs, was enriched for H3K27ac in endothelial cells and myeloid cells, but not in fibroblasts (*SI Appendix*, Fig. S4*C*). These data suggest that *Csf1* expression is regulated by cell type-specific enhancers, and that density-dependent control of *Csf1* by YAP1 is a feature of fibroblasts.

### Fibroblasts Produce CSF1 to Support Macrophage Populations.

Production of CSF1 from local tissues is essential for the maintenance, proliferation, and differentiation of tissue-resident macrophages (20, 21). Our previous work has demonstrated that fibroblasts produce CSF1 to support growth and survival of macrophages (18). Similar communication has been observed between fibroblasts and tissue-resident macrophages in vivo (30, 31, 51). These communication circuits are essential to maintain homeostasis of macrophage numbers. By modeling the paracrine communication between macrophages and fibroblasts (28, 52), we predict that changes in the expression of *Csf1* in fibroblasts affect the homeostatic number of macrophages as well as the relative ratio between macrophages and fibroblasts (*SI Appendix*, Fig. S4*D*). To test whether fibroblasts are a physiologically relevant source of CSF1 for macrophages in vivo, we used a genetic model of CSF1-deficiency in PDGFRα^+^ cells, including various fibroblast populations such as hepatic stellate cells. In the livers of PDGFRα^Cre^*Csf1*^fl/fl^ mice, we observed a decrease in macrophage frequency both by flow cytometry and histological analysis (*SI Appendix*, Fig. S4 *E* and *F*). These data demonstrate that fibroblast populations provide an important source of CSF1 for macrophages in vivo.

In order to determine whether YAP1 regulation of *Csf1* expression in fibroblasts can directly control macrophage numbers, we returned to our previously established macrophage-fibroblast in vitro coculture system in combination with YAP1^CA^ fibroblasts. As expected, constitutive activation of YAP1 resulted in an increase in fibroblast numbers, consistent with a role for Hippo-YAP signaling in autonomous control of cell proliferation (Fig. 4*F*). In addition, macrophage numbers, as well as the overall macrophage to fibroblast ratio, significantly increased when YAP1 was active (Fig. 4*F*). With elevated expression of *Csf1*, macrophages and fibroblasts still exhibited a stable ratio regardless of starting conditions (Fig. 4*G*). This feature of stability was consistent with our previous observations and theoretical model prediction. It also demonstrated that YAP1-mediated regulation of *Csf1* is sufficient to control the number of macrophages in a defined compartment.

Like most growth factors, CSF1 acts in the vicinity of its source, thereby affecting both local numbers and spatial arrangement of macrophages (21). We employed an agent-based modeling approach to gain insights into whether density-dependent CSF1 production impacts the spatial distribution of macrophages (*SI Appendix*). The agent-based modeling simulations revealed an intriguing role of density-dependent production of paracrine growth factors in regulating macrophages in space, which may impact the spatial organization of macrophages in complex tissues (*SI Appendix*, Fig. S5).

### Space Limitation Regulates Gene Expression through Actin-Dependent Mechanisms.

The Hippo-YAP and TGF-β pathways are well known to be involved in sensing cellular environment (6, 53). YAP activity can be regulated by mechanical properties and composition of the ECM. TGF-β signaling can be induced by soluble and contact-dependent signals. How these signaling pathways are induced by space availability remains elusive. We first tested whether the density-dependent expression programs in fibroblasts were due to soluble signals or responses to ECM components. We transferred supernatants of low-density cultures to high-density cells, and vice versa, to test whether fibroblasts sense density signals through soluble factors (*SI Appendix*, Fig. S6*A*). We also transferred cells onto decellularized ECM, from low- or high-density cells, to test whether fibroblasts sense density through properties of the ECM (*SI Appendix*, Fig. S6*B*). However, neither supernatant transfer nor ECM “transfer” mimicked the effect of either low- or high-density conditions. Although these experiments do not exclude the role of ECM, they suggest that fibroblasts may sense cell density or space availability through additional, cell-intrinsic mechanisms.

**Fig. 5.**
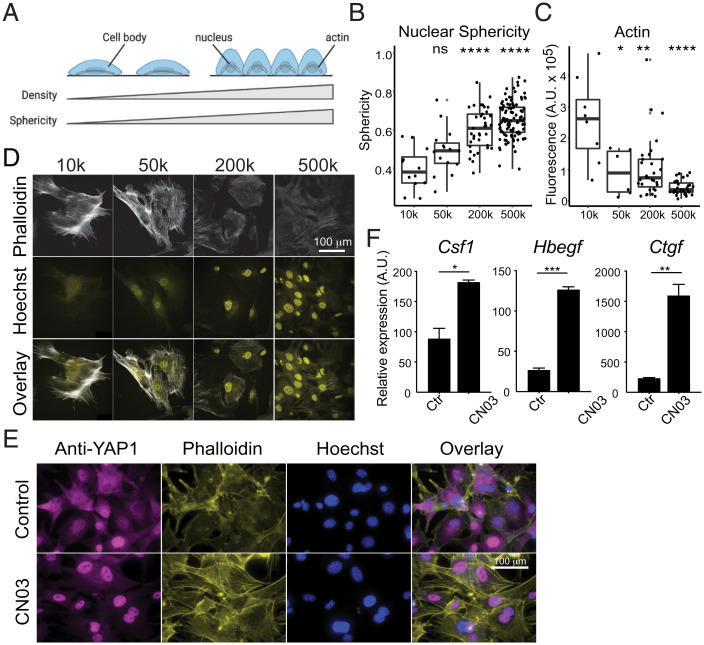
Actin-dependent mechanisms regulate density gene expression. (*A*) Diagram depicting the relationship between increased nuclear sphericity and increased cell density. (*B*) Quantification of nuclear sphericity of MEFs, calculated as the ratio between the length and width of a nucleus. Each point represents an individual nucleus. Six images per density were quantified. (*C*) Confocal images of immunofluorescent staining of actin filaments (phalloidin) in MEFs at different cell densities. (*D*) Quantification of fluorescence intensity of actin staining at different cell densities. Fluorescence intensity of actin inside the cell is corrected by the background. Each data point represents one cell. (*E*) Representative immunofluorescence images of YAP1 localization in MEFs after 4 h treatment with CN03. (*F*) Growth factor expression in MEFs after 4 h treatment with Rho activator (CN03 peptide). Wilcox *t* test. ns, *P* > 0.05, *P < 0.05, **P < 0.01, ***P < 0.001, ****P < 0.0001.

We observed that the average surface area of a cell is smaller at high density than that of a cell at low density (*SI Appendix*, Fig. S6 *C* and *D*). The length and width of nuclei are also smaller at high cell density than at low cell density (*SI Appendix*, Fig. S6 *E* and *F*), while the height or “thickness” of nuclei shows the opposite trend (*SI Appendix*, Fig. S6*G*), and similar nuclear volume is maintained across conditions (*SI Appendix*, Fig. S6*H*). We reasoned that lack of contact with neighboring cells allows expansion of the cell body and thus flattens the nucleus (Fig. 5*A*). To examine the changes in nuclear shape, we quantified the sphericity of nuclei using the ratio between the length and the width (the longest and second longest axes of nuclei, respectively). A spherical nucleus has a ratio close to 1 while a flattened nucleus has a much smaller ratio. Indeed, nuclei in cells at low cell density are significantly less spherical than in cells at high density (Fig. 5*B*), indicating that nuclei at low and high cell densities experience different mechanical pressure.

Previously, it was reported that stiff matrices promote nucleation of actin fibers that apply pressure to the nucleus, leading to YAP1 nuclear translocation (54, 55). In our experiments, discrete actin fibers were commonly observed at low density (Fig. 5*C*) and displayed higher fluorescent intensity (Fig. 5*D*). We thus tested if formation of actin fibers directly regulates the expression of density-dependent growth factors. CN03, a peptide derived from bacterial deamidase toxins, activates RhoA by locking it into a constitutively active form (56). RhoA then activates Rho activated protein kinase (ROCK), which promotes actin polymerization through LIM kinase and coffilin, and contractility of actin fibers through activation of myosin-light chain kinase and inhibition of myosin-light chain phosphatase (57–59). Following 2 h of treatment with CN03, we observed that actin filaments formed across the length of cells, and that YAP1 was found almost exclusively in the nucleus even at high cell density (Fig. 5*E*). Interestingly, activation of RhoA strongly induced the expression *Csf1* and other YAP1-dependent growth factors, including *Bdnf* and *Ereg* (Fig. 4*F* and *SI Appendix*, Fig. S7*A*), and siRNA knockdown of *Yap1* abolished the RhoA-dependent activation of these genes (*SI Appendix*, Fig. S7*A*).

To further test if actin assembly is responsible for elevated *Csf1* expression when cells grow at low density, we treated low-density cells with either Y27632 (60), a ROCK inhibitor, or Latrunculin A (61), an inhibitor of actin assembly. We found that blocking the formation of actin filaments was sufficient to reduce nuclear localization of YAP1 and expression of *Csf1* (*SI Appendix*, Fig. S7 *B* and *C*). In addition to YAP1-regulated growth factors, two growth factors that are strongly dependent on cell density and TGF-β signaling, *Hbegf* and *Ctgf*, are also activated by RhoA, but independent of YAP1 (Fig. 5*E* and *SI Appendix*, Fig. S7*A*). Indeed, treatment of CN03 triggers significant enrichment of SMAD2/3 in the nucleus, and this localization can be reversed by inhibiting actin assembly with Y27632 (*SI Appendix*, Fig. S7*D*). Both Y27632 and Latrunculin A can reduce the expression of *Ctgf* and *Hbegf* in cells cultured at low density (*SI Appendix*, Fig. S7*B*). Moreover, at the transcriptomic level, density-dependent expression programs are highly enriched for genes that are regulated by Rho signaling (MsigDB, “Berenjeno transformed by RhoA”, *P* < 10^−114^). Altogether, these data demonstrated that the activity of both YAP1 and SMAD can be regulated by actin assembly. Given the distinct change of nuclear shape at different cell densities, these data suggest that sensing space availability may be dependent on RhoA signaling, and that density-dependent expression programs may be controlled through mechanical forces acting on the nucleus.

### Environmental Conditions Regulate Growth Factor Expression.

**Fig. 6.**
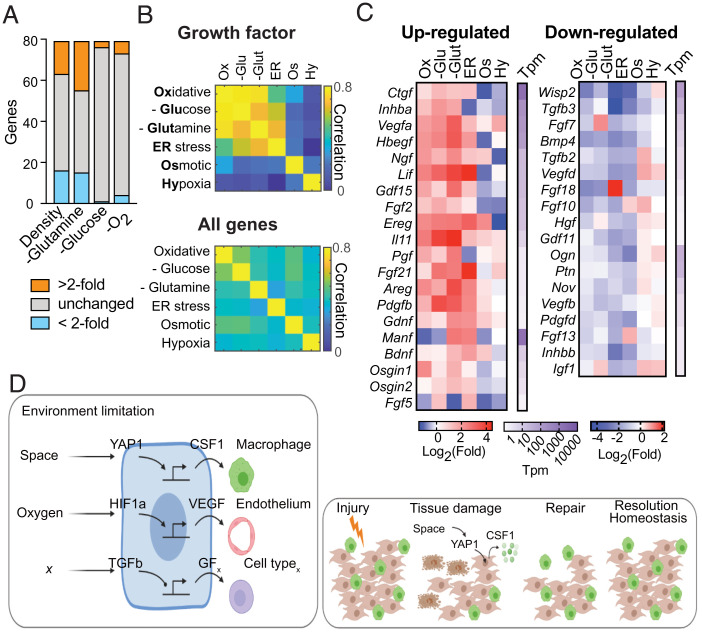
External environment regulates growth factor expression in fibroblasts. (*A*) The number of growth factors differentially regulated in MEFs under different densities and nutrient-depleted conditions. (*B*) Heatmap showing the correlation of growth factor genes or the complete transcriptome among different conditions. (*C*) Heatmap showing expression fold-changes of selected growth factors, grouped as commonly induced or repressed in MEFs at different conditions. (*D*) Sensing of environmental conditions including space and oxygen regulates growth factor production through conserved signaling pathways such as Hippo-YAP (*Left*). These mechanisms may explain how cell loss due to tissue damage is repaired and tissue homeostasis restored (*Right*).

In ecology, extrinsic environmental factors determine the carrying capacity, or maximum size, of a given population. In a tissue compartment, space is one such limiting factor. We demonstrated that the cell type sensitive to space limitation regulates the expression of growth factors for a cell type that is insensitive to space constraints. A variety of other environmental variables, including nutrient or oxygen levels, could limit the number of cells in a compartment. We thus screened expression of growth factors in fibroblasts under diverse conditions, including amino acid deprivation, glucose deprivation, and hypoxia. Several growth factors were strongly induced or repressed by each condition (Fig. 6*A*). To test if there is a pattern in the control of growth factors, we included additional environmental conditions that may have effects on carrying capacity such as oxidative, osmotic, and endoplasmic reticulum (ER) stress. We analyzed how changes in expression vary among different conditions in comparison to the overall change at the transcriptomic level. Surprisingly, transcriptional changes of growth factor genes were highly correlated among oxidative stress, ER stress, and glucose and glutamine deprivation (Fig. 6*B*). In contrast, the overall cellular responses to these stress conditions and nutrient limitations showed poor correlation (Fig. 6*B*).

These analyses suggested that a subset of growth factors is regulated by factors of cellular environment. For example, *Ctgf*, *Vegfa*, *Hbegf*, *Lif*, *Gdf15*, *Il11*, and *Areg* are among the growth factors that are commonly induced, while *Bmp4*, *Vegfd*, *Hgf*, and *Pdgfd* are among the growth factors that are commonly repressed under these conditions (Fig. 6*C*). Altogether, these data reveal a central role for environmental sensing by fibroblasts in the control of growth factor production.

## Discussion

The population sizes of different cell types within a tissue compartment must be tightly regulated to ensure proper tissue functioning, prevent overgrowth, and allow for regeneration and repair (Fig. 6*D*). Two modes of cell number control have been described previously: one is based on space availability, which can be mediated through either cell–cell or cell–ECM contact; the other is based on growth factor availability (2, 7, 15). However, whether these control strategies function independently from each other is not known. Here we demonstrated that fibroblasts and macrophages, two universal cell types within tissues, each use a different strategy to control their numbers. Fibroblast proliferation is more sensitive to the availability of space, while macrophage expansion is highly sensitive to the availability of a growth factor. Moreover, we found that production of the macrophage-specific growth factor CSF1 by fibroblasts was directly regulated by Hippo-YAP signaling in response to space limitation. This coupling between space availability with growth factor production provides a simple link between the two different modes of cell number control (Fig. 6*D*).

The Hippo-YAP pathway is known to regulate cell proliferation, apoptosis, stemness, and differentiation in response to a variety of internal and external signals (11, 12, 24, 41, 45). Therefore, its role in controlling cell density-dependent responses was not unexpected. However, its involvement in the regulation of growth factor expression was intriguing because this pathway has been most commonly described for cell autonomous (or cell-type autonomous) responses. However, in a recent study, YAP was found to not only inhibit pluripotency of stem cells autonomously, but to also induce pluripotency of neighboring cells through production of matrix proteins (62). This work, alongside our findings, suggests that Hippo-YAP1 signaling also has important functions in regulating cell non-autonomous responses to environmental cues through cellular communication. Moreover, we found that changes in cell and nuclear shape that occur in sparse versus dense environments may reflect mechanical pressure of the actin cytoskeleton on the nucleus, which could in turn regulate translocation of YAP1 into the nucleus. Interestingly, we found that manipulation of RhoA signaling and assembly of actin can regulate density-dependent gene expression that is dependent on both YAP1 and SMAD. Recently, it has been shown that pressure on the nucleus regulates cell migration behavior (63, 64). Our work suggests that the nucleus can be a mechanical sensor for cell density and control both cell autonomous and cell non-autonomous proliferation. This is consistent with recent findings demonstrating the role of the cell nucleus in mechanosensing (63–64) (65). While our data suggest that changes in nuclear shape are downstream of cytoskeletal alterations driving YAP1, this connection may involve a combination of physical inputs and not changes in nuclear shape alone (66). Additional work will be necessary to assess the direct link between changes in nuclear shape and YAP1 activity.

Signaling pathways that sense tissue microenvironment are often not specific to a particular cell type. For example, epithelial cells, fibroblasts, stem cells, and hepatocytes all employ Hippo-YAP1 signaling pathways to control their proliferation in response to cell–cell and cell–ECM interactions (11, 12, 24, 41). We found that YAP1 regulates the expression of *Csf1* via a conserved distal enhancer that is uniquely active in fibroblasts. This Hippo-YAP1–regulated enhancer thus couples fibroblast density with macrophage numbers. This observation suggests that certain cells within tissues may have specialized functions in regulating tissue composition in response to different environmental factors (Fig. 6*D*). For example, macrophages are well known to sense hypoxia through HIF1α and in turn secrete VEGFA to promote endothelial growth and vascularization (67). Skin macrophages sense hyperosmolarity and produce VEGFC to enhance proliferation of lymphatic endothelial cells (68). This link between environmental sensing by one cell type and growth factor production for another cell type may be a general feature employed to regulate tissue composition. Cell type-specific regulatory elements of growth factor gene expression may provide molecular fingerprints to uncover the underlying links between tissue microenvironment and cell type composition.

Not all cell types respond to growth factors or environmental signals by increasing rates of proliferation. Indeed, cells can be categorized as labile, stable, or permanent, based on their proliferative capacity. Permanent cells, such as neurons and cardiomyocytes, have limited proliferative capacity and their numbers in adults are primarily determined during development. Labile cells, such as intestinal epithelial cells, skin keratinocytes, and most hematopoietic cell types, are continuously replenished from stem cells and have high proliferative capacity until they reach a terminally differentiated state, at which point they become postmitotic. The appropriate ratios and numbers of cells going through different developmental stages can be in principle determined based on negative feedback between differentiated cells and their progenitors (69, 70). Finally, stable cells, such as macrophages, fibroblasts, and hepatocytes, are normally quiescent but can proliferate even in a fully differentiated state in response to specific growth factors (35, 71, 72). Through studying macrophages and fibroblasts, we found that population size control of two different stable cell types is coordinated: the signaling pathway that limits proliferation of one cell type regulates expansion of the other cell type. Tissue-resident immune cells, including mast cells, macrophages, and innate lymphoid cells, are distinct “stable” cell types and their numbers are likely regulated in a similar fashion, by signals from stromal cells such as fibroblasts.

In an ecosystem, carrying capacity is defined as the maximum population size that can be supported in a given environment (4). In tissues in vivo or cell cultures in vitro, many variables can regulate cellular proliferation and survival, but carrying capacity is determined by the variables that are the most limiting for growth. At standard culture conditions, we found that fibroblasts and macrophages use different strategies of cell number control. The mechanism used by fibroblasts to detect changes in space availability directly controls growth factor production for macrophages. Fibroblasts are well known to be regulated by growth factors, such as PDGFs and EGFs. However, the compartment size for fibroblasts at steady state is primarily determined by space availability. Additionally, we found that changes in other environmental factors, such as oxygen or nutrient levels, regulated growth factor production in fibroblasts. In this way, unique environmental stimuli, such as mechanical cues, oxygen, or nutrients, determine the levels of growth factors produced by fibroblasts for macrophages and other cell types (Fig. 6*D*). Whether this is a general mechanism of growth factor regulation in other cell types that respond to limitations in space remains to be determined.

Finally, our findings may also have implications in the progression of “tissue-level” diseases, such as cancer and fibrosis. One conserved feature of tumors, regardless of anatomical location or tissue of origin, is dysregulation of cell composition and escape from normal growth control mechanisms. Increased YAP/TAZ activity in tumor cells is one of the mechanisms linked to cancer initiation and progression. In fact, YAP/TAZ activation is observed in a broad spectrum of human cancers, leading to tumor cell proliferation, survival, and invasion (73). Additionally, YAP/TAZ up-regulation in cancer-associated fibroblasts is associated with high-grade tumors and poor prognosis via effects on ECM stiffness (74). Our data suggest there may be an additional tumor-promoting function of YAP1 activation: the up-regulation of growth factors within the tumor microenvironment. Whether YAP1 activity in cancer-associated fibroblasts drives the production of CSF1 or other growth factors is an important open question. Similarly, YAP/TAZ hyperactivation is observed in both the epithelial and fibroblast tissue compartments of fibrotic lesions (75). This association with fibrosis has largely been attributed to YAP1-dependent transcription of ECM remodeling genes and feed-forward enhancement of ECM stiffness and contractile actin formation (76). Indeed, we found that actin polymerization promotes YAP1 nuclear translocation and downstream growth factor production. The extent to which YAP1 control of CSF1 also contributes to fibrotic disease is not yet known. A more detailed understanding of the upstream signals driving nuclear YAP1 will have important implications for the normalization of transformed and/or fibrotic tissues.

### Limitations and Caveats.

While this study focused on the proof-of-concept and molecular mechanisms of growth factor production, it remains to be determined how different factors of the cell microenvironment regulate growth factor production in different cell types in vivo. The contribution of ECM versus cell density as primary indicators of space availability within tissues needs to be further defined. Addressing these questions will require development of in vivo models that allow for evaluation of ECM properties as well as spatially resolved dynamic monitoring of growth factor expression.

## Materials and Methods

### Mice.

C57BL/6J (stock #000664), Pdgfra-cre (stock #013148), and Tgfbr2^fl/fl^ (stock #012603) mice were purchased from the Jackson Laboratory. YapKI^fl/fl^ mice were generated previously (24). Csf1^fl/fl^ mice were generously provided by Sherry Abboud Werner (University of Texas Health Science Center, San Antonio, TX). All mice were maintained in a specific pathogen-free facility and animal experimentation was conducted in accordance with Yale University School of Medicine institutional guidelines.

### Cell Culture and Differentiation.

BMDMs were differentiated from whole bone marrow from female mice (8- to 12-wk old) in the presence of L929-conditioned media. MEFs were harvested from male and female embryonic day 13.5 to 14.5 embryos and sorted for purity. All cell cultures were maintained in a 37 °C incubator at 5% CO_2_. See *SI Appendix* for detailed information for cell isolation, purification, and culturing.

### Cytokines and Chemicals.

Growth factors were used at the indicated concentration. If not specified, CSF-1 and PDGF-BB were used at 50 ng/mL, TGF-β was used at 10 ng/mL, and WNT3A was used at 50 ng/mL. Chemical inhibitors were titrated based on published literature and used at the following concentrations: Rho activator CN03 2 μg/mL, TGF-β pathway inhibitor LY364947 1 μM, actin filament inhibitor LatA 500 nM, Rock inhibitor Y27632 10 μM. For experiments with chemical inhibitors, cells were plated at different cell densities overnight, treated with inhibitors, and collected at the indicated time for analyses of RNA expression, cellular signaling, or immunofluorescent imaging. For cells treated with CN03, all conditions were plated in complete DMEM first, then changed to serum-free complete DMEM overnight before applying CN03.

### Viral Transduction.

Expression vectors pMigR1-Cre/IRES-GFP (Cre-GFP) or pMigR1-IRES-GFP (GFP) were cotransfected into 293T cells with retrovirus packaging vector pCL-Eco using the Lipofectamine 2000 kit (Thermo Fisher Scientific, #11668019) according to the manufacturer’s instructions. After 24 h, cell culture medium was changed to complete DMEM. After 24 h, viral supernatant was collected, filtered through 70-μm cell filters and directly applied to low-passage unsorted MEFs, at 1:1 ratio to the existing medium. Successfully transduced MEFs were sorted based on GFP-positivity and allowed to rest for at least one passage before performing coculture experiments.

### Immunofluorescence Imaging.

Cells were cultured in eight-well chamber slides and fixed in 4% paraformaldehyde and permeabilized using 0.1% saponin in blocking buffer (HBSS containing 3% BSA, 0.2% gelatin, and 0.02% NaN_3_) and stained with rabbit anti-mouse YAP1 (Cell Signaling Technology, D8H1X), FITC-conjugated phalloidin, and goat anti-rabbit IgG (H+L) Alexa Fluor 594 secondary (ThermoFisher, A-11007). Hoechst 33342 was used to stain nuclei. Cells were mounted on microscope slides with ProLong Diamond Antifade Mountant (Molecular Probes). Imaging was performed with Leica AF6000 Modular System (fluorescent microscope) or Leica SP8 (confocal microscope). Confocal imaging was acquired using HC PL APO CS2 63×/1.40 oil objective and the 405-nm and Argon (488-nm) lasers collected on different sequentials.

### Gene Silencing Using siRNA.

P1-P2 unsorted MEFs were lifted from plates using 0.05% Trypsin + EDTA. Each siRNA was incubated with OptiMEM while RNAiMax was incubated with OptiMEM for 5 min at room temperature. The two solutions were mixed together dropwise and incubated for 20 min. siRNA mixtures were then plated in 12-well plates and 50,000 MEFs were added to each well. All siRNAs were used at a final concentration of 5 nmol. Cells were harvested and RNA isolated after 3 d of incubation with siRNA.

### RNA Isolation and qRT-PCR.

RNA was purified from cells using Qiagen RNeasy columns with on-column DNase digestion according to the manufacturer’s instructions. cDNA was reverse-transcribed with MMLV reverse transcriptase (Clontech) using oligo-dT_20_ primers. qRT-PCR was performed on a CFX96 Real-Time System (Bio-Rad) using PerfeCTa SYBR Green SuperMix (Quanta Biosciences). Relative expression units were calculated as transcript levels of target genes over 1/1,000 of Actb. Primers used for qRT-PCR are listed in *SI Appendix*, Table S1.

### RNA-Seq Analysis.

Illumina fastq files were downloaded from Illumina Basespace and were aligned with Kallisto program with default settings (77) against all cDNA transcripts in mouse genome annotation GRCm38 (ftp://ftp.ensembl.org/pub/release-90/fasta/mus_musculus/cdna/). The ENSEMBL IDs of each cDNA transcript were matched to the official gene symbols through BioaRt in R. The expression of each transcript is expressed in TPM. When multiple transcripts match to the same gene, the expression of the gene is calculated by summing the TPM of all matched transcripts.

### ChIP-Seq Analysis.

Reads from Illumina paired-end fastq files were mapped to the mouse genome (mm9) using Bowtie (78) with the options “-S -q –best -m 1 -p 20 -v 1 -a -strata” to generate SAM files. Read duplicates were removed and BAM files were generated with the Picard toolkit (http://broadinstitute.github.io/picard). Bigwig files were generated using Homer software (79) using the option “-fsize 1e20” and uploaded to the University of California, Santa Cruz genome browser. Enriched ChIP regions were identified using MACS2 (80) using the options “'-f BAMPE –bw 200 -B -g mm”. Transcription factor motifs in the enriched ChIP regions were identified also using Homer software using the options “-mask -size 200 -mis 3 -S 30 -len 8,10,12”. Genes were associated with the enriched chip regions by locating the closest gene also using Homer software.

### Analysis of Histone Modifications.

Histone modification (H3K4me1, H3K4me3, K3K27ac) data of MEFs were obtained from Mouse Encode http://www.mouseencode.org/. H3K27ac data of human skeletal muscle fibroblasts (HSMM), endothelial cells (HUVEC), myeloid leukemia cells (K562), B-lymphoblastoid cells (GM12878), stem cells (hESC), epidermal keratinocytes (NHEK), and human lung fibroblasts (NHLF) were obtained from the human ENCODE project (https://www.encodeproject.org/). The tracks of HSMM, HUVEC, and K562 were shown as examples.

## Supplementary Material

Supplementary File

## Data Availability

All high-throughput sequencing data generated from this study have been deposited in a publicly accessible database. RNA sequencing data of fibroblasts at different cell densities and at various stress conditions from this study have been deposited to the Gene Expression Omnibus (GEO) database, https://www.ncbi.nlm.nih.gov/geo [accession nos. PRJNA719850 (81) and GSE205381 (82)]. ChIP-seq data of endogenous YAP1 MEFs have been deposited to the GEO [accession no. GSE184774 (83)]. Single-cell RNA-seq data of human lung fibroblasts were obtained from the GEO [accession no. GSE136831 (84)]. ChIP-seq data of SMAD3 in MEFs were obtained from GSE85177. H3K27ac, H3K4me, and H3K4me3 of MEFs were obtained from GSM1000139 (85), GSM769028 (86), and GSM769029 (87), respectively (88). Human H2K27ac of fibroblasts, endothelial cells, and myeloid leukemia were obtained from GSM733755 (89), GSM733691 (90), and GSM733656 (91). Customized scripts for density-dependent analysis, clustering in Matlab, imaging quantification in R, and circuit modeling in Mathematica are available upon reasonable request. The *SI Appendix* includes detailed methods for cell culture, flow cytometry, cell quantification, Western blot, imaging analysis and quantification, ECM and supernatant transfer, cell isolation from liver, RNA-seq, ChIP-seq, density-dependent differential expression analysis, single-cell RNA-seq analysis, and agent-based modeling.
