# Supplementary material for: Microenvironmental sensing by fibroblasts controls macrophage population size
Source: Proc Natl Acad Sci U S A. 2022 Aug 5;119(32):e2205360119. doi: 10.1073/pnas.2205360119 (PMC9371703; doi:10.1073/pnas.2205360119)
Supplement: Supplementary File [file pnas.2205360119.sapp.pdf]

PNAS

Supplementary Information for

Microenvironmental Sensing by Fibroblasts Controls Macrophage Population Size

Xu Zhou,* Ruth A. Franklin,* Miri Adler, Trevor S. Carter, Emily Condiff, Taylor S. Adams, Scott D. Pope, Naomi H. Philip, Matthew L. Meizlish, Naftali Kaminski and Ruslan Medzhitov

* These authors contributed equally

Corresponding author: Ruslan Medzhitov
Email: Ruslan.Medzhitov@yale.edu

This PDF file includes:

Supplementary text
Supplementary methods
Figures S1 to S6
SI References

Supplementary Information Text

Agent-based Modeling of the MPs FBs circuit

We developed an agent-based modeling approach to explore how density-dependent growth factor production affects the spatial distribution of the cells. Agent-based modeling is a computational approach for simulating the behavior of multiple agents (cells in this case) that interact with each other according to specified rules (1, 2). In the simulations, macrophages and fibroblasts are represented as red and blue cells respectively, and obey the following rules: (1) red cells produce a paracrine growth signal (*B1*, e.g. PDGFb) for blue cells, (2) blue cells produce a paracrine growth signal (*R*, e.g. CSF1) for red cells and an autocrine growth signal (*B2*, e.g. PDGFa/HBEGF) (3), (3) every cell computes the total amount of growth factor in its vicinity (tunable parameter), and undergoes one of three fate choices: die, stay, or divide, depending on the amount of available growth factor (Figure S5A). The threshold values for cell death, survival, and proliferation are

tunable parameters of the system. To model the density-dependent regulation of CSF1, we considered that the production of signal *R* from a blue cell is a decreasing function of the overall blue cell density in its vicinity. This model recapitulates the bi-stability of the two-cell circuit (3, 4), where the steady-state of the system depends on the initial conditions of these two populations (Figure S5B-C). However, the impact of density-dependent regulation on the spatial distribution of red and blue cells is mild and sensitive to the choice of parameter values. Tissues are often composed of much more than two cell types. To test if the density-dependent regulation may have an oversized role in conditions of complex cell composition, we introduced a third 'inert' cell type (or a non-cell structure) that takes up space but does not interact with either blue or red cells via growth factors (Figure S5A, lime cell). We considered several arbitrary structures of lime cells and presented the simulation where lime cells are organized as rings of different radius. Without density-dependence, the steady-state spatial pattern of red and blue cells is uniformly distributed throughout (Figure S5D). Interestingly, with density-dependence, red cells localize away from the lime cells, as if the red cells are excluded by the lime cells. This effect is even more striking in the center of small lime cell rings, where the loss of red cells impacts the survival of accompanied blue cells, eventually creating an empty zone (Figure S5E). This may represent conditions where the emergence of non-cellular structure or growth of 'inert' cells exclude macrophages and fibroblasts (such as in late-stage fibrosis).

Supplementary methods

Cell culture and differentiation

Bone marrow-derived macrophages (BMDMs) were differentiated from whole bone marrow from female mice (8 to 12 weeks old) in the presence of L929-conditioned media. Femurs and tibias were removed from mice, flushed, and exposed to hypotonic lysis (ACK lysing buffer, ThermoFisher) to remove red blood cells. Cells were plated in macrophage growth media (MGM) overnight (RPMI + 2 mM L-glutamine, 1 mM sodium pyruvate, 10 mM HEPES, 200 U/mL penicillin/streptomycin, 10% FBS, and 30% L929-conditioned media). The next day (day 1), nonadherent cells were harvested, washed, and replated in 15 cm Petri dishes in 20 mL of MGM. On day 4, 15 mL of MGM was added to the plate. On Day 6-7, cells were lifted with 3 mM cold EDTA in PBS for 15 min and plated at appropriate concentrations for coculturing. All cell cultures were maintained in a 37°C incubator at 5% CO2.

Mouse embryonic fibroblasts (MEFs) were harvested from male and female E13.5-E14.5 embryos and sorted for purity. Staged embryos were removed from a pregnant female by removing the uterus and separating each embryo from its amniotic sac. The head and ''red tissue,'' including fetal liver, were removed and discarded. If genotyping was required, the head served as the source of DNA and embryos were kept separated. The remaining portion of each embryo was minced

using razor blades in 0.05% trypsin + EDTA and placed in a 37°C incubator for 30 min. After digestion, the tissue was transferred into a conical tube, washed with complete DMEM (DMEM + 2 mM L-glutamine, 1 mM sodium pyruvate, 10 mM HEPES, 200 U/mL penicillin/streptomycin, 10% FBS; GIBCO) and resuspended in complete DMEM in 15 cm tissue culture plates overnight. The following day, cells and undigested tissue debris were lifted from the plates using 0.05% trypsin + EDTA, spun down, resuspended, and filtered over a 70 μm filter. These cells were expanded for 1-2 passages and then sorted for CD45-, CD11b-, and F4/80-negativity to exclude contaminating macrophages. The sorted MEFs were split once after sorting to allow for recovery and used for experiments at p4-p7. Unsorted MEFs (p1-p5) were used in experiments where noted.

Flow cytometry for cell quantification

MEFs were harvested from tissue culture plates by incubation with 0.05% Trypsin + EDTA. BMDMs were harvested from non-tissue culture treated plates by incubation with 3mM EDTA in PBS. MEFs and BMDMs in co-culture were harvested first by incubation with 0.05% Trypsin + EDTA, and next by cell scraping to remove any remaining attached cells. All cells were washed and transferred to round-bottom 96-well plates for FACS staining. Fluorochrome-conjugated antibodies against CD45 (clone 30-F11), CD11b (M1/70), and F4/80 (BM8) were purchased from eBioscience/ThermoFisher. Dead cells were excluded using ThermoFisher LIVE/DEAD Fixable Aqua Dead Cell Stain and binding to Fc receptors was blocked using CD16/CD32 (clone 93, eBioscience/ThermoFisher). Absolute cell numbers were calculated using counting beads (123count eBeads, ThermoFisher), which were added following harvest, immediately prior to running samples on the flow cytometer (50 uL of beads were added to 150 uL sample). Absolute cell numbers were calculated according to manufacturer's instructions, using the following equation: ((# of live single cells acquired * 0.05)/(# of beads acquired * 0.15)) * eBead concentration. To correct for cell loss during harvest, we applied a standard curve adjustment based on the original cell number plated and the bead-calculated cell number. All samples were acquired on a Becton Dickinson LSR II Flow cytometer and analyzed using FlowJo.

Western blot analysis

Cells were washed 1X with ice cold PBS before harvesting. Protein was extracted in 1x SDS buffer (62.5 mM Tris-HCl, pH 6.8, 2% SDS, 25% glycerol, 0.01% bromophenol blue) on ice followed by heating at 95˚C for 10 minutes. Multiple wells of low density cells were combined during collection to reach similar total cell numbers at each cell density. Lysates corresponding to equal numbers of cells were loaded into 4-20% TGX Precast gels (Biorad) and ran with Tris/glycine running buffer (Biorad). Protein was transferred onto activated PVDF membrane (Millipore) using trans-blot Turbo system (Biorad), and then blocked using 5% BSA in TBST (20 mM Tris, 150 mM NaCl, 0.05% Tween 20) for at least 1 hour at room temperature. Primary antibody was incubated in 5% BSA in TBST at 4˚C overnight at recommended dilution (anti-SMAD2/3 D7G7 1:1000, anti-phospho-

SMAD2/3 D27F4 1:1000, anti-GAPDH 1:2000), and secondary antibody was incubated in TBST at room temperature for 30 min at 1:5000. Samples were washed 3x with TBST following each round of antibody incubation. Protein was visualized using ECL western blotting substrate (ThermoScientific) and autoradiography film. Densitometry was calculated using ImageJ software. Band intensity of interest was normalized to GAPDH for each sample, and averaged across biological replicates.

Assay to quantify growth sensitivity to space availability and growth factor

We used Click-iT Plus EdU Flow Cytometry Assay Kit to quantify proliferation of MEFs or BMDMs at different conditions. Briefly, MEF or BMDM monocultures were plated in 6-well tissue culture plates 1 day prior to EdU incorporation assay. EdU (10 mM) was added to the cells for 2 hr and the cells were harvested as above. Cells were first stained with surface antibodies and then EdU incorporation was detected using the Click-iT Plus EdU Flow Cytometry Assay Kit according to the manufacturer's instructions (ThermoFisher). Samples were then acquired on a Becton Dickinson LSR II Flow cytometer and analyzed using FlowJo. For analysis of sensitivity to space availability, cells were plated at 50,000 to 1,000,000 per well in 6-well plates, which covers the range that proliferation changes linearly with cell density. In each experiment, the percentage of EdU^+ cells was plotted against cell density, and the slope of linear regression was normalized to represent change of proliferation per 100,000 cells (ΔEdu+/100K). For analysis of sensitivity to growth factors, we treated cells with 0 - 20 ng/ml growth factors overnight, before analyzing using the EdU assay kit described above. To confirm the observed results were not due to insufficient growth signals, particularly for fibroblasts, cells were treated with high concentrations of CSF-1 and PDGF-BB up to 200 ng/ml. The proliferative effect of recombinant growth factors saturated at less than 10 ng/ml for both macrophages or fibroblasts. To quantify the sensitivity to growth factor, we calculated the max growth difference between control (without recombinant growth factor) and growth factor stimulated conditions. Multiple experiments were averaged to infer the sensitivity of growth to space availability or growth factors, where each dot represents one experiment.

ECM and supernatant transfer

For ECM "transfer," 200,000 unsorted MEFs were plated per well in 6 well plates in 10% FBS for 2 days. After 2 days, cells were removed using 25 mM EDTA in PBS and blasting by pipetting. Plates were then washed twice with PBS and fresh 20,000 or 200,000 MEFs were plated on top of decellularized matrix. Cells were harvested and RNA isolated after overnight culture. For supernatant transfer experiments, supernatant from either high density (200,000) or low density (25,000) MEF cultures was removed after 12 hours, spun down to remove any cellular debris, and added to MEFs plated at low density (25,000). Cells were harvested and RNA isolated after overnight culture.

Cell isolation from liver

Livers from sacrificed mice were prepared by mechanical disruption, followed by 30 minute treatment with 2 mg/ml Collagenase Type 4 (Worthington Biochemical) in PBS at 37°C with continuous shaking. Digested tissues were mashed through 70 μm filters, layered in a 33% and 66% Percoll gradient (Sigma), and centrifuged at 3000 rpm for 30 min without brake. Cells at the interface were collected and analyzed by flow cytometry. Fluorochrome-conjugated antibodies against CD45 (clone 30-F11), CD64 (clone X54-5/7.1), and MerTK (clone DS5MMER) were purchased from eBioscience/ThermoFisher. Dead cells were excluded using Zombie Yellow Fixable Viability Stain (BioLegend) and binding to Fc receptors was blocked using CD16/CD32 (clone 93, eBioscience/ThermoFisher). Cell numbers were calculated using counting beads (123count eBeads, ThermoFisher) as described above.

Quantification of nuclear shape and actin fluorescence

Slides stained with phalloidin and DAPI were imaged with a Phalloidin corrected fluorescence intensity measured and calculated using Fiji (ImageJ) version 2.1.0/1.53c.(5) Cells were manually outlined using freehand selection around phalloidin staining. For each cell, area, mean gray value, and integrated density were collected for all cells and an area with no cells was collected for background signal quantification. Corrected fluorescence intensity calculated with the equation: Integrated density – (area of cell * mean gray value of background). Figures generated using ggplot2 in R with R Studio. Statistics calculated using Wilcox t test comparing all densities to the 10k density individually.

Nucleus quantification was obtained after collecting confocal z stacks with slices every 0.3um encompassing the entire nucleus of all cells fully within the field of view. 3D projections viewed using Imaris software (Bitplane), and surfaces were generated using DAPI fluorescence intensity. Statistics for all surfaces were exported and figures were generated using ggplot2 in R with R Studio. Length, width, and thickness were obtained using the object oriented measurements with bounding box length A, B, and C respectively. Statistics calculated in R using Wilcox t test comparing all densities to the 10k density individually.

RNA sequencing

Primary p0 MEFs were expanded for 1 passage and sorted to remove contaminating cells. Sorted MEFs were culture for 1 passage and plated at the following cell density 10,000/well (2,000/cm^2), 50,000/well (5,000/cm^2), 200,000/well (21,000/cm^2), and 500,000/well (52,000/cm^2) in DMEM. The highest cell density is close to the theoretical carrying capacity that we determined previously (3). After overnight culture, adherent cells were washed twice with ice-cold PBS and collected with RLT buffer (Qiagen RNeasy kit). RNA was purified from cells using Qiagen RNeasy columns with on-column DNAse digestion according to the manufacturer's instructions. Sequencing libraries were constructed following Illumina Tru-seq stranded mRNA protocol. Paired-end sequencing was

performed with Next-seq 500 with either 76 or 38 bp from each end. Two sets of replicated densities were obtained from different batches of primary MEFs.

For analyzing transcriptional response of MEFs in different stress conditions, 100,000/well (10,000/cm^2) MEFs were plated in DMEM. For oxidative stress or osmotic stress, 10 μM sodium arsenite (Sigma Aldrich) or 100 mM NaCl (American Bio) were added to the cells after overnight culture for 3 hours. For ER stress, 1 μM thapsigargin (Cayman) was added for 10 hours after overnight culture. For glucose depletion, glutamine depletion and hypoxic conditions, after overnight culture following the initial seeding, cells were incubated in glucose-free DMEM (Gibco), glutamine-free DMEM (Gibco), or placed in a hypoxia chamber at 0.1% oxygen for 10 hours. Cell collection, RNA purification, and sequencing library construction were carried out as described above.

Differential expression analysis

Differential expression analysis was performed using sleuth program (6). This analysis returned 34475 genes in total in the mouse genome. Using an adjusted p-value of 0.05, we identified 3046 genes that are significantly differentially expressed between any two cell densities and have an expression higher than 2 TPM. To further identify genes that are differentially expressed biologically, we further selected genes that are activated or repressed more than 1.5-fold. Primary fibroblast cultures have significant biological variations between each preparation. To identify the genes consistently influenced by cell density, we performed the experiments with two separately isolated batches of MEFs. As expected, there are significant variations between biological replicates at baseline. We developed an automated correlation-based method to identify the genes that change with cell density consistently. To select a threshold for defining consistency between biological replicates, we computationally shuffled the data to generate a randomized background with no logical connection between each density for each gene. After ranking the genes from the least correlated to the best correlated, we calculated the accumulated gene counts as a function of the correlation coefficient. For the randomized dataset, this gives a linear line, because different degrees of correlation happen by chance (Figure S1C). For the experimental dataset, this gives a curve skewed towards high correlation coefficient (Figure S1C). We used the intersection between the randomized and experimental data curves as the threshold for selecting genes with consistent changes in expression. Intuitively, far more truly correlated genes are selected than genes with randomized expression at this correlation coefficient. Following these criteria, we identified 1950 genes as density-dependent genes. All fold changes are calculated using a pseudo count of 1 TPM to avoid dividing expression value by 0 and small expression values. These genes were then clustered using K-mean clustering based on log2(fold) changes in TPM expression. K-mean clustering was performed with customized codes using built-in functions in Matlab, until the clustering converged in 20 iterations. Clusters of 2 - 10 were screened and 7 was identified as the minimum number of clusters to characterize the general trend of expression changes at different

cell densities. To identify genes differentially regulated at various stress conditions, we selected genes that are either induced or repressed by more than 2-fold comparing to the control condition.

Chromatin-immunoprecipitation and high-throughput sequencing

MEFs were crosslinked by adding formaldehyde to the medium to a final concentration of 1% in 15 cm plates with continuous, gentle rocking at RT for 10 minutes. The reaction was quenched by adding 125 mM final concentration of glycine, with continuous, gentle rocking at RT for 5 minutes. The cells were washed 3 times with ice cold PBS, scraped with 10 ml of PBS, and spun down for 5 minutes at 1,500 rpm at 4°C. For Chromatin-immunoprecipitation, for cell pellets of 10^6 cells, 1 ml cell lysis buffer (10 mM Hepes pH 7.3, 85 mM KCl, 1 mM EDTA, 0.5% IGEPAL CA-630, 1x protease inhibitors (ThermoFisher Halt)) was used to resuspend the pellet and incubated on ice for 5 minutes. The lysate was centrifuged for 5 minutes at 4,000 rpm at 4°C, the supernatant was removed, and the pellet was resuspended in 0.3 ml of nuclear lysis buffer for sonication (10mM Tris pH 8.0, 0.5% N-lauroylsarcosine, 0.1% sodium deoxycholate, 100 mM NaCl, 1 mM EDTA, 0.5 mM EGTA, 1x protease inhibitors (ThermoFisher Halt)). Cell lysate was transferred to 1.5 ml Bioruptor Plus TPX microtube (Diagenode) and was sonicated for 4 rounds of 15 cycles of 30 seconds on/30 seconds off on high power (Diagenode Biorupter plus). After sonication, the chromatin was centrifuged for 15 minutes at max speed at 4°C and the supernatant was carefully transferred to a new tube and 1% Triton X-100 was added. 0.7 mL of ChIP dilution buffer (20 mM Tris pH 7.5, 0.5% Triton X-100, 100 mM NaCl, 1 mM EDTA, 1x protease inhibitor) was then added to the sonicated chromatin.

10% of the chromatin was taken as “Input”, and the remaining chromatin incubated overnight with anti-YAP1 antibody at 1:50 dilution (CST) overnight on a rotator at 4°C. Protein G Dynabeads (ThermoFisher 10004D) were washed 3 times with PBS + 0.5% BSA and then added to the chromatin and antibody mixture and rotated at 4°C for 3 hours. Protein G dynabeads were washed sequentially with low-salt wash buffer (20 mM Tris pH 7.5, 0.1% SDS, 1 % Triton X-100, 150 mM NaCl, 1 mM EDTA), and LiCl wash buffer (10 mM Tris pH 7.5, 1% sodium deoxycholate, 1% Triton X-100, 250 mM LiCl, 1 mM EDTA) 3 times, and washed once with TE to remove detergents and salts. Bound DNA was eluted twice by resuspending the beads with 125 ul of elution buffer (50 mM $NaHCO_3$, 1% SDS) and incubated for 10 minutes at RT, followed by 3 minutes of vigorous shaking at 37°C. Eluted DNA was digested with proteinase K at 55°C for 2 hours and reverse-crosslinked at 65°C overnight. DNA was purified using the Qiagen MinElute PCR Purification Kit and ChIPseq libraries were generated using the NEBNext Ultra II DNA Library Prep Kit for Illumina. Sequencing was performed with Illumina NextSeq500 for paired-end 38bp reads.

Sequence motif and function enrichment analysis

Clusters of genes were organized into different groups and analyzed with Homer using motifs.pl. For sequence motif enrichment, the analysis focused on gene promoter regions, defined by -1000

and +200bp from transcription start site, searching for DNA sequence motifs from 6 to 10 bp in length. For function enrichment analysis, annotated cellular signaling pathways were extracted from Kegg pathways and all significantly enriched terms ($p <= 0.01$) were curated based on enrichment statistics.

Modeling the effect of CSF1 production rate on macrophage numbers

To model the effect of varying the production rate of CSF1 in fibroblasts, we used mathematical models describing cell circuit communication between macrophages and fibroblasts developed in our previous work (3, 4). Model parameters are as described before except the rate of CSF1 production. We numerically solved the ODE system to extract the steady-state level of macrophage and fibroblast numbers at different CSF1 production rates. This is performed with the NDSolveValue and FindRoot functions in Mathematica 12.1.1.0.

Single cell analysis

We analyzed single-cell RNA-seq data of human lung fibroblasts from Adams et al..(7) The data includes 1051, 786 and 4329 cells from control, COPD, and IPF patients, respectively. To preprocess the data, we used 'Sanity' – a recently developed method to normalize single-cell data and to infer the transcriptional activity of genes. Sanity is a unique Bayesian procedure for normalizing single-cell RNA-seq data from first principles.(8) Following the Sanity normalization, for each comparison between datasets, we removed genes with low expression ($\log_{10}$(average expression) > -11) and low expression variance (standard deviation > 0.05) across single cells. In total, 6934 genes in control fibroblasts, 7011 genes in integrated control and COPD fibroblasts, and 7376 genes in integrated control and IPF fibroblasts passed the selection thresholds. For the control fibroblasts, we observed a fraction of cells with extremely low gene expression ($\log_{10}$(average expression) < -9.9). After removing these outlier cells, the control samples included 945 cells for downstream analysis.

After preprocessing the data, we first established landmark genes whose expression depends on density of cells and shows significant variability in the single cell dataset. We considered z-scored expression in the single cell datasets for the top density dependent genes that show more than 2-fold differential expression between low (10K) and high (500K) cell densities. We identified 17 landmark genes for the control samples, including *Serpine1*, which is representative of genes expressed at a higher level at low cell density, and DCN, representative of genes expressed at a higher level at high cell density. Since the relative expression of these landmark genes correlates with the density of cells, we then defined a density score for each cell as the difference between the average expression of the high-density genes and the average expression of the low-density genes. For example, if a cell expresses high-density genes at a higher level than low-density genes, it will be given a high score for high density, and vice versa. We next sorted the cells according to their density scores and inferred the genes that are highly variable across this density axis. We

repeated this analysis for the control fibroblasts alone, the control and COPD fibroblasts, and the control and IPF fibroblasts.

Agent-based Modeling

The agent-based modeling simulation was built in NetLogo, a programming language and IDE developed by Uri Wilensky and Northwestern University. In these simulations, we utilize the Patch type, which represents an individual square on the grid of the simulation space, entirely made of Patches. Blue patches emit a red signal (*R*, promoting red proliferation) and a self-promoting blue signal (B_2). Red patches emit a blue signal (B_1, promoting blue proliferation). At each timestep, the patches calculate the value of the signal perceived in their neighborhood. A patch's neighborhood is defined as a circular radius centered around the patch itself and is a parameter that can be varied. The simulations presented in Figure S5 were run with a neighborhood radius of five patches. The calculation of signals for each patch at each timestep is given by the following equations.

With density-dependent regulation of red signal: $R_{i,t+1} = R_{i,t} + N_B \frac{R}{N_B+N_L+K}$, where $R_{i,t+1}$ is the amount of red signal perceived by patch *i* at timestep t, N_B and N_L are the number of blue and lime patches in the neighborhood of patch *i*, R is the amount of red signal produced per patch, and K is a parameter that controls the effect of the density-dependence of blue cells on the red signal.

Without density-dependent regulation of red signal: $R_{i,t+1} = R_{i,t} + N_B R$. Since blue signal is not subjected to density-dependent regulation, the amount of blue signal perceived by the patches at each timestep is the same in both cases: $B_{i,t+1} = B_{i,t} + N_R B_1 + N_B B_2$.

Depending on the value of signal, the patch decides to divide, stay the same, or die. In case of division, the patch randomly selects an empty (white) neighboring patch and changes it to be the same color. In the case of cell death, the patch turns empty (white), allowing for other neighboring cells to divide into its space.

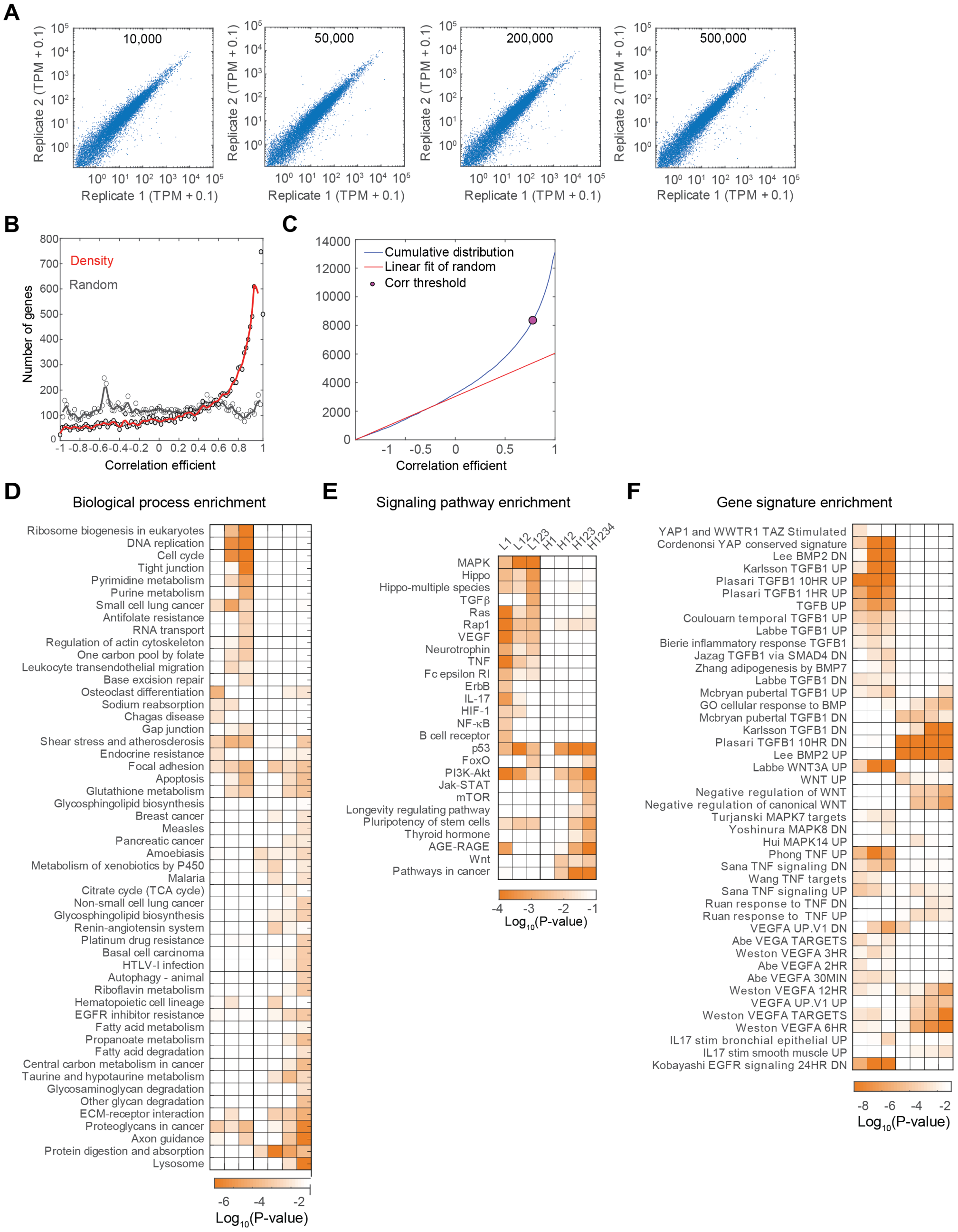

Figure S1. Transcriptional analyses of density-dependent expression programs

A. Transcriptome correlation of biological replicates at the indicated cell density. 0.1 TPM is added to each gene.

B. Distribution of gene-specific correlation coefficients between two sets of randomized RNAseq data or biological replicates at matched cell densities. Expression data were generated randomly using a bootstrap method by shuffling genes and samples. Correlation coefficients of all genes are binned every 0.02 unit, and the sliding-window average is shown as colored curve.

C. Cumulative distribution of the coefficients between two sets of biological replicates. Linear fit was generated using the distribution of coefficients that are less than 0. Linear fit of the cumulative distribution of correlation coefficients calculated using randomized

data set is shown in red. The pink circle marks the threshold for consistent density regulation.

D. Enrichment of biological processes in groups of genes induced at low or high density.
E. Enrichment of signaling pathways in groups of genes induced at low or high density.
F. Enrichment of molecular signature in groups of genes induced at low or high density.

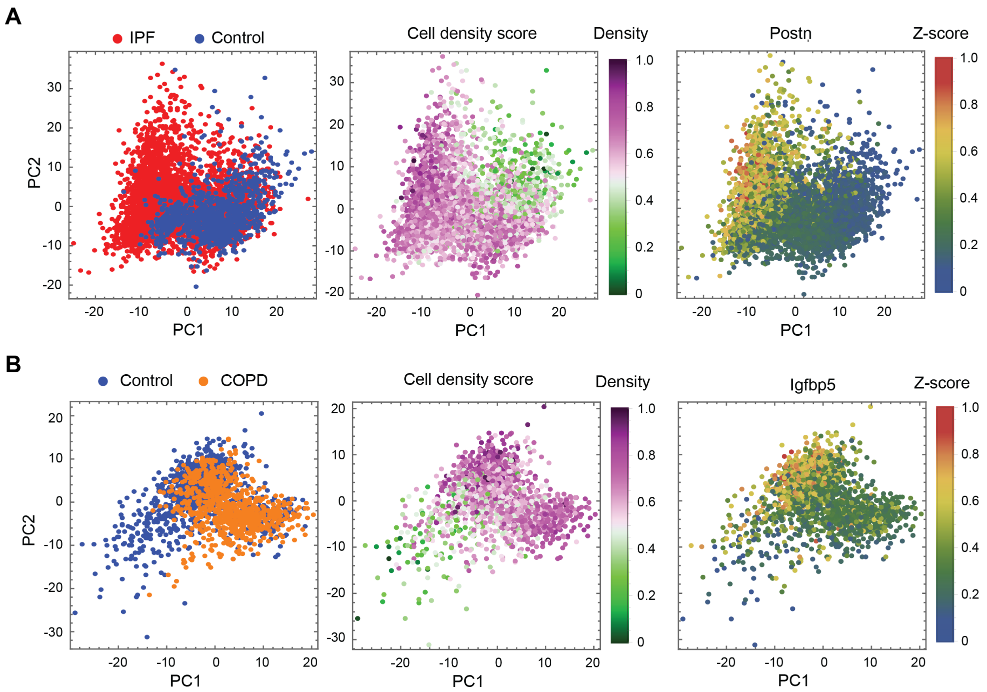

Figure S2. Single-cell reconstruction based on landmark genes reveals new density-dependent genes.

A. Single-cell RNA-seq (scRNA-seq) data of fibroblasts from control (blue) and IPF (red) lungs projected on the first two principal components (left panel). Cells are colored by their density score, defined by the average expression of landmark density dependent genes (middle panel). POSTN is highly expressed in cells with high density scores (right panel).
B. Same as A but with fibroblasts from COPD lungs in orange (left panel), and expression of IGFP5 (right panel).

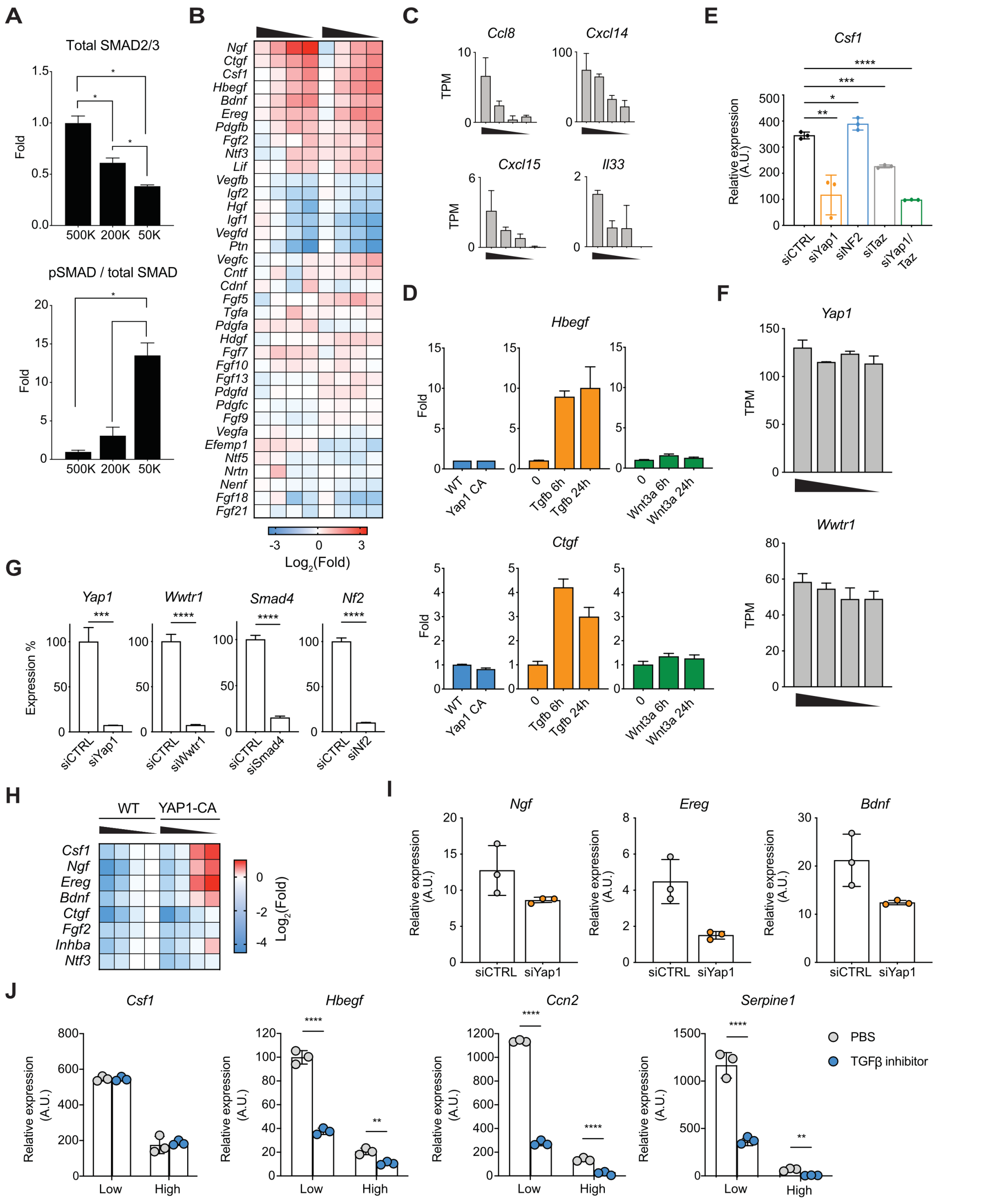

Figure S3. YAP1-dependent control of growth factor expression

A. Quantification of total Smad2/3 proteins, and the ratio between phosphorylated and unphosphorylated SMAD proteins at different MEF densities as measured by Western blot. Cells were analyzed after overnight culture at the specified cell densities.
B. Heatmap showing the expression of growth factors in MEFs at different densities. Growth factors with TPM > 5 are shown.
C. Expression of chemokines and cytokines significantly regulated by density (high to low density).
D. *Hbegf and Ctgf* expression in Yap^{CA} MEFs plated overnight, or MEFs treated with recombinant Tgfb or Wnt3a for 6 or 24 hours.

E. *Csf1* expression in MEFs 3 days after transduction with siRNAs targeting *Yap1*, *NF2*, or *Wwtr1* (Taz) alone, *Yap1* and *Wwtr1*z together, or scrambled control siRNA (siCTRL).
F. *Yap1* and *Wwtr1* expression in MEFs plated at decreasing cell densities, calculated as TPM from RNAseq data.
G. siRNA knock-down of *Yap1*, *Taz*, *NF2* and *Smad4* 3 days after siRNA transduction.
H. Heatmap showing RT-qPCR quantified expression of selected growth factors at different cell densities in control and Yap^{CA} MEFs.
I. RNA expression of *Ngf*, *Ereg*, and *Bdnf* 3 days after transduction with siRNAs targeting *Yap1*.
J. RNA expression in MEFs cultured at low or high density after 4 hours treatment with 1uM TGF-β inhibitor, LY364947.

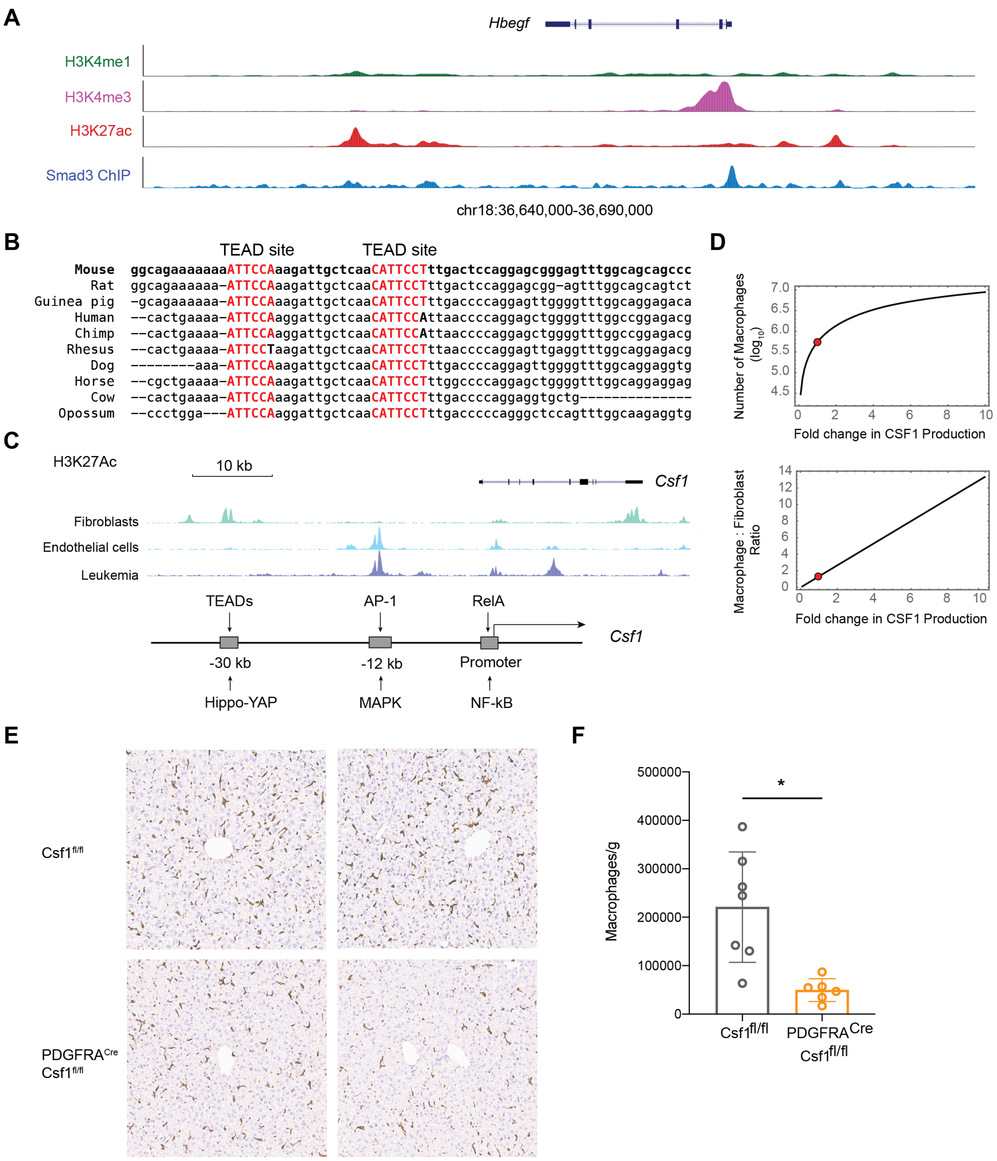

Figure S4. Regulatory elements at the *Csf1* and *Hbegf* loci and control of macrophage numbers by stromal cells

A. Genomic tracks displaying ChIP-seq of histone modifications (H3K27ac, H3K4me1, and H3K4me3) and Smad3 binding at *Hbegf* gene locus in MEFs.
B. Alignment of genomic sequences within *Csf1* enhancer showing conservation of TEAD binding sequences among mammalian species.
C. ChIP-seq tracks of H3K27ac in human cells (top) and illustration of enhancers and promoter at *Csf1* locus. H3K27ac data were obtained from the human ENCODE project. Diagram illustrates the known signals that regulate Csf1 expression (bottom).
D. Cell circuit modeling prediction of macrophage numbers and macrophage to fibroblast ratios as a function of CSF1 production rate. The red circles mark the data measured using wild type cells.
E. Representative immunofluorescence images of F4/80 staining (brown) in liver sections of $Csf1^{fl/fl}$ and $Pdgfra^{Cre}Csf1^{fl/fl}$ mice.
F. Number of liver macrophages in $Csf1^{fl/fl}$ and $Pdgfra^{Cre}Csf1^{fl/fl}$ mice per gram of liver tissue. Cells are gated on live $CD45^{+}CD64^{+}MerTK^{+}$ cells.

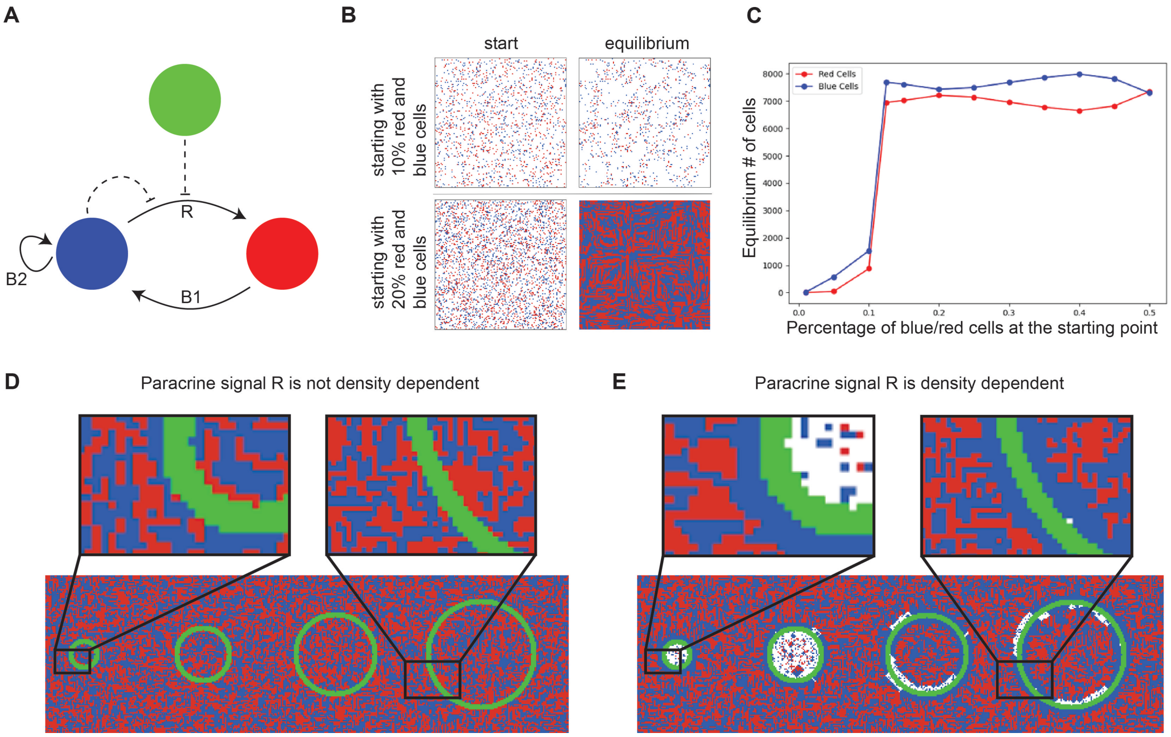

Figure S5. Agent-based model of the macrophage-fibroblast cell circuit reveals impact on spatial organization.

A. Schematics of agent-based modeling describing the interactions between MPs and FBs. Blue cells, FBs; red cells, MPs; lime cells, “inert” cells.
B. Representative simulation of the two-cell circuit in 2D. Two snapshots were taken at the start and at equilibrium for each simulation, at initial conditions with low cell density (upper panels) and high cell density (lower panels).
C. Graph showing the steady-state cell number as function of the initial density of blue and red cells.

D, E. Representative images of simulation with a third cell type, without (D) and with (E) density-dependent regulation of paracrine growth factor *R*. Lime, inert cells or non-cell structure.

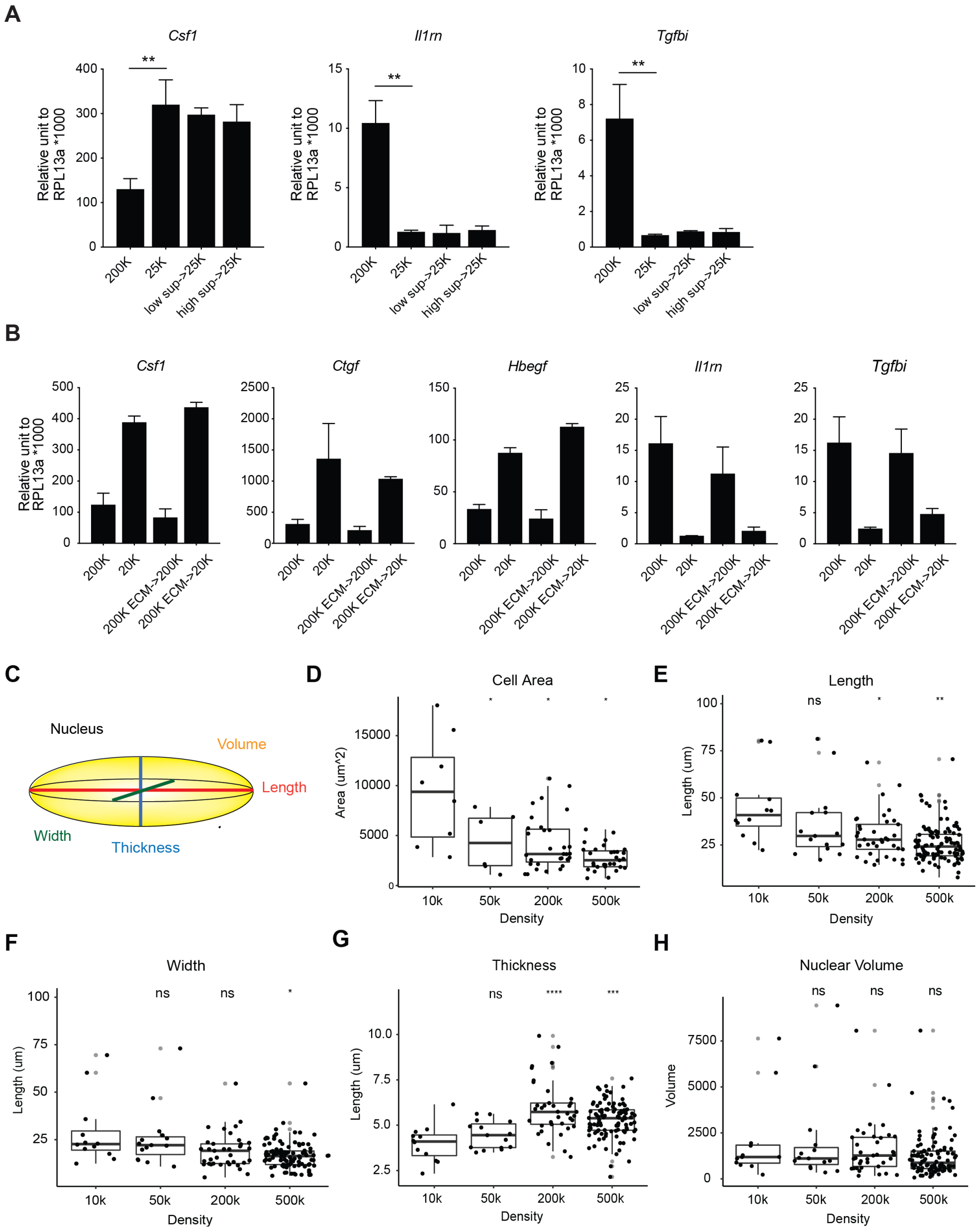

Figure S6. Cellular mechanisms controlling density-dependent expression programs

A. Expression of growth factors in MEFs plated at low or high density and cultured overnight in conditioned supernatant. Two-tailed t-test was applied to all statistical analyses, *p<0.05, **p<0.01.

B. Expression of growth factors in MEFs plated on decellularized extracellular matrix from low or high density cell cultures.

C. Graphical representation of the measurements for size and shape of nuclei.

D-H. Quantification of cross-sectional cell area (**D**), length (**E**), width (**F**), thickness (**G**) and volume (**H**) of nuclei. Nuclear length is measured as the longest axis of the nucleus and nuclear width is quantified along the axis perpendicular to nuclear length in the *xy* plane. Nuclear thickness is defined as the shortest axis of the nucleus, typically along the z axis. Each point represents one nucleus. Statistical significance is performed with Wilcox t test. ns p>0.05, * p<0.05, ** p<0.01, *** p<0.001, **** p<0.0001.

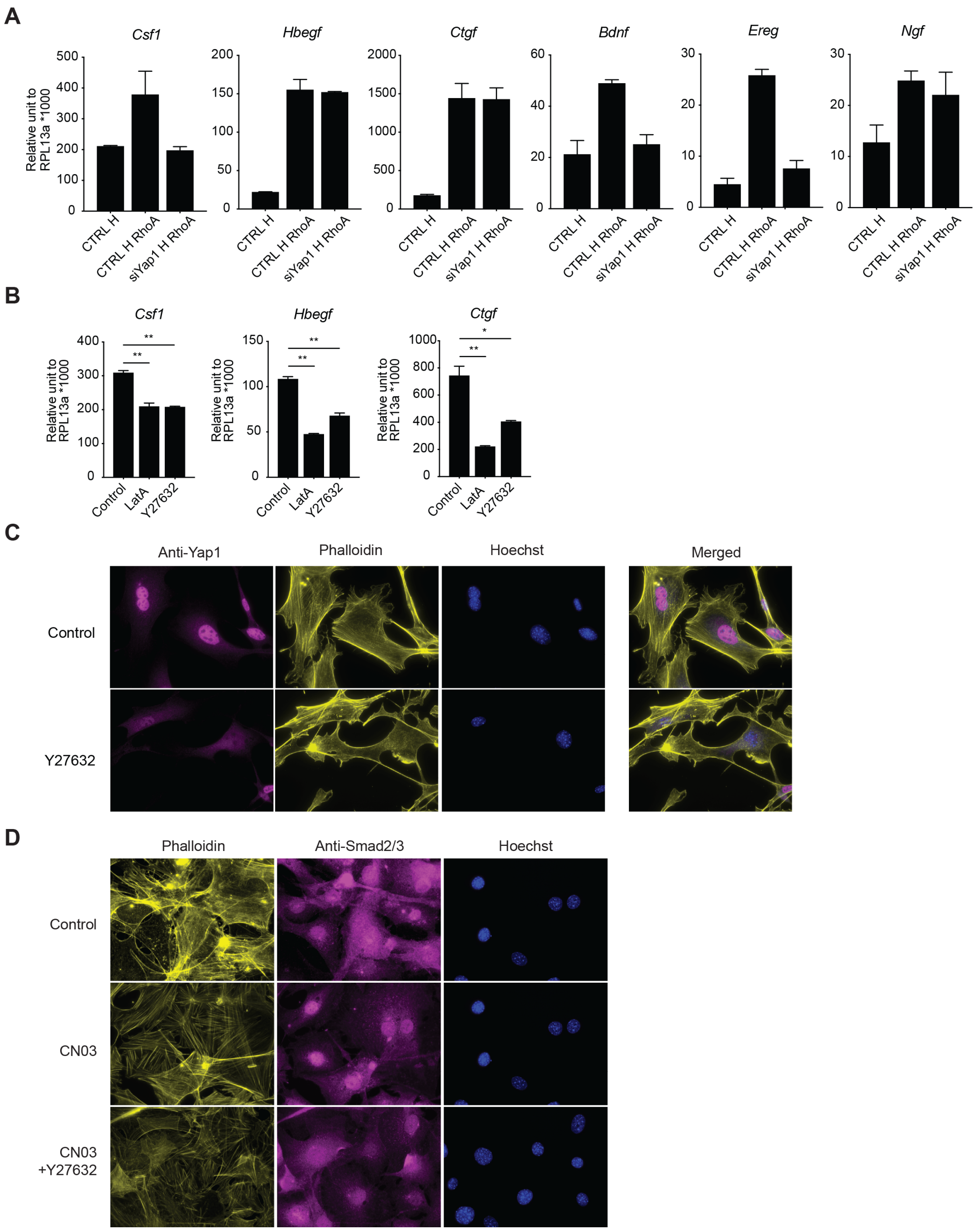

Figure S7. Actin-dependent mechanisms regulate activation of YAP1 and SMADs

A. Growth factor expression in MEFs plated at high cell density after treatment with RhoA activator (CN03 peptide) for 4 hours with or without 3 day transduction with siRNA targeting *Yap1* prior to peptide treatment.
B. Expression of growth factors in MEFs treated with Latrunculin A (LatA) or ROCK inhibitor (Y27632) for 4 hours.
C. Localization of YAP1 in MEFs plated at low density (equivalent to 50,000/well) and treated with Y27632 for 4 hours.
D. Localization of SMAD proteins (total Smad2/3) in MEFs plated at high cell density (equivalent to 500,000/well) and treated with RhoA activator (CN03 peptide) or RhoA activator in combination with ROCK inhibitor for 4 hours.

SI References

1. C. M. Glen, M. L. Kemp, E. O. Voit, Agent-based modeling of morphogenetic systems: Advantages and challenges. *PLOS Computational Biology* **15**, e1006577 (2019).
2. U. Wilensky, W. Rand, An introduction to agent-based modeling Modeling Natural, Social, and Engineered Complex Systems with NetLogo. *Agent analyst*, 1e30 (2013).
3. X. Zhou, *et al.*, Circuit Design Features of a Stable Two-Cell System. *Cell* **172**, 744-757.e17 (2018).
4. M. Adler, *et al.*, Endocytosis as a stabilizing mechanism for tissue homeostasis. *Proceedings of the National Academy of Sciences* **115**, E1926–E1935 (2018).
5. J. Schindelin, *et al.*, Fiji: An open-source platform for biological-image analysis. *Nature Methods* **9**, 676–682 (2012).
6. H. Pimentel, N. L. Bray, S. Puente, P. Melsted, L. Pachter, Differential analysis of RNA-seq incorporating quantification uncertainty. *Nature Methods* **14** (2017).
7. T. S. Adams, *et al.*, Single-cell RNA-seq reveals ectopic and aberrant lung-resident cell populations in idiopathic pulmonary fibrosis. *Science Advances* **6** (2020).
8. J. Breda, M. Zavolan, E. van Nimwegen, Bayesian inference of the gene expression states of single cells from scRNA-seq data. *bioRxiv*, 2019.12.28.889956 (2019).
